# Beyond Density: Unveiling the Trade‐Off in Catalytic Site Optimization Using Defect‐Engineered UiO‐66

**DOI:** 10.1002/advs.202510578

**Published:** 2025-09-02

**Authors:** Guo‐Ying Han, Yi Ji, Xiang‐Yu Li, Yu Gai, Guo‐Zhen Hou, Qin‐Yi Cheng, Zhong Zhang, Yiwei Liu, Jia Wang, Pascal Van Der Voort, Guangjin Hou, Xin‐Ping Wu, Gaohong He, Xiao Feng

**Affiliations:** ^1^ School of Chemistry Dalian University of Technology Dalian 116024 China; ^2^ State Key Laboratory of Catalysis Dalian National Laboratory for Clean Energy 2011‐Collaborative Innovation Center of Chemistry for Energy Materials Dalian Institute of Chemical Physics Chinese Academy of Sciences Dalian 116023 China; ^3^ State Key Laboratory of Green Chemical Engineering and Industrial Catalysis Centre for Computational Chemistry and Research Institute of Industrial Catalysis School of Chemistry and Molecular Engineering East China University of Science and Technology Shanghai 200237 China; ^4^ Center for Ordered Materials, Organometallics and Catalysis (COMOC), Department of Chemistry Ghent University Ghent B‐9000 Belgium; ^5^ State Key Laboratory of Fine Chemicals Frontiers Science Center for Smart Materials School of Chemical Engineering Dalian University of Technology Dalian 116024 China

**Keywords:** catalytic sites density, cluster defects, defect types, linker defects, MOFs

## Abstract

Enhancing intrinsic activity and increasing catalytic site density are two widely employed strategies to improve catalytic performance. Although typically considered independently, their interplay remains poorly understood. Here, two UiO‐66 metal–organic frameworks (MOFs) with distinct catalytic site densities—linker‐defective UiO‐66L and cluster‐defective UiO‐66C—are synthesized and systematically compared. Despite a higher density of open Zr catalytic sites, UiO‐66L exhibited lower catalytic activity than UiO‐66C across four model reactions, performing similarly to defect‐free UiO‐66. Although defect engineering is expected to enlarge pore connectivity, diffusion‐ordered spectroscopy (DOSY) and molecular dynamics (MD) simulations surprisingly reveal that UiO‐66C exhibits similar diffusion rates to defect‐free UiO‐66, while UiO‐66L shows significantly slower diffusion. This discrepancy is attributed to self‐adsorption of reactants at the high‐density catalytic sites, which induces local diffusion resistance even in the presence of expanded channels. These findings reveal a performance trade‐off between catalytic site density and intrinsic activity, establishing a critical threshold beyond which further increases in site density can hinder rather than enhance catalysis.

## Graphical abstract

A well‐designed experimental model reveals a trade‐off between intrinsic activity and catalytic site density in performance optimization, due to inherent diffusion resistance arising from substrate adsorption at catalytic sites.

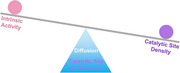

## 1 Introduction

Increasing catalytic performance is often achieved by increasing the density of catalytic sites and/or improving the intrinsic activity of each site.^[^ ^1^, ^2^, ^3^, ^4^, ^5^, ^6^, ^7^, ^8^ ^]^ Jaramillo and co‐workers have highlighted these two strategies as fundamental principles for designing catalysts.^[^ ^1^ ^]^ Metal‐organic frameworks (MOFs) serve as a model platform for catalytic studies, owing to their well‐defined structural characteristics and highly dispersed catalytic sites.^[^ ^9^, ^10^, ^11^, ^12^, ^13^, ^14^, ^15^, ^16^ ^]^ Based on these design principles, strategies such as metal adjustment,^[^ ^17^, ^18^, ^19^, ^20^, ^21^ ^]^ linker modification,^[^ ^22^, ^23^, ^24^ ^]^ uniform pore loading,^[^ ^25^, ^26^, ^27^, ^28^, ^29^ ^]^ or defect engineering^[^ ^30^, ^31^, ^32^, ^33^, ^34^ ^]^ have been employed to boost catalytic performance. Schuhmann et al. introduced defects into Cu‐triazole frameworks to create open metal sites, thereby enhancing the catalytic performance of Cu‐triazole and effectively tuning the selectivity of the electrocatalytic CO_2_ reduction reaction.^[^ ^35^ ^]^ Liu et al. demonstrated that the substitution of Zr with Ce enhanced the intrinsic metal activity, resulting in optimal hydrolysis performance.^[^ ^36^ ^]^ A critical question arises: Do these design principles have an upper limit in enhancing catalytic activity?

**Figure 1:**
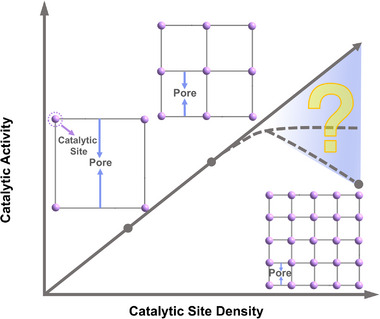
The relationship between the catalytic site density and catalytic activity.

When mass transport is not the rate‐limiting step, enhancing intrinsic activity and increasing the density of active sites are generally regarded as effective strategies to boost catalytic performance. Liu et al. theoretically demonstrated that increasing the density of catalytic active sites does not necessarily lead to a higher reaction rate, due to the aggregation of produced gas molecules near the catalytic sites.^[^ ^37^ ^]^ Heenen et al. further revealed that mesoscopic mass transport effects can influence catalytic selectivity.^[^ ^38^ ^]^ Additionally, when catalytic sites are brought into close proximity, their mutual interactions may alter their behavior.^[^ ^39^, ^40^ ^]^ These observations raise a fundamental question: Is a higher density of catalytic sites always the optimal solution in the absence of external mass transport diffusion resistance (**Figure** **1**)? Moreover, is there an inherent limit to the effectiveness of these two design principles?

**Figure 2:**
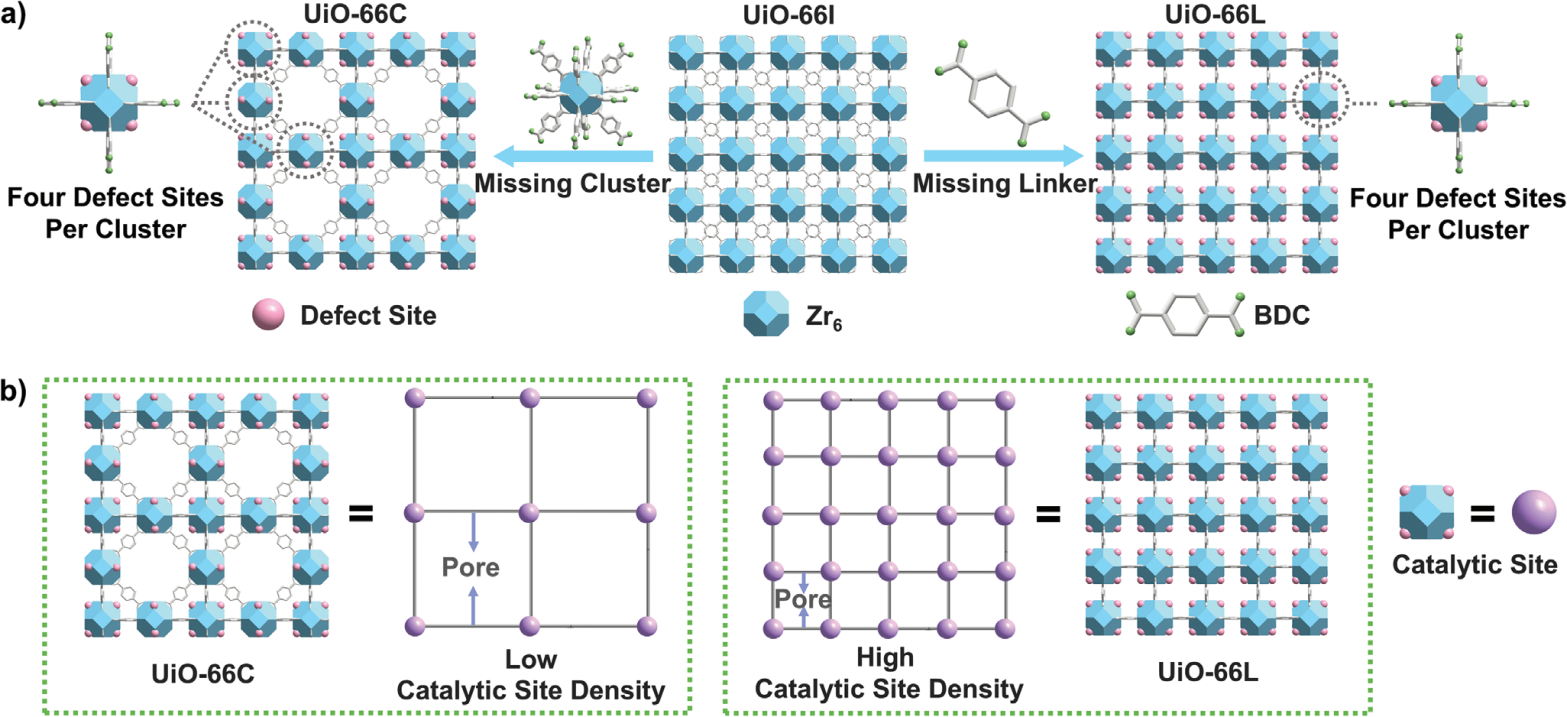
a) The structural differences between defect‐free UiO‐66 and UiO‐66 with linker or cluster defects, where illustrations show that different defect types maintain the same number of open metal sites per cluster. b) Schematic representation of catalytic site density for two defect types UiO‐66.

Developing well‐defined experimental model systems to clarify this relationship poses a considerable challenge. In this work, UiO‐66 MOFs with distinct types of defects were precisely synthesized using a pre‐introduction and selective etching strategy (**Figure** **2** and Figure S1, Supporting Information), including UiO‐66L, which features the missing linker, and UiO‐66C, with the missing cluster. This approach overcomes the challenge of simultaneously producing both linker and cluster defects, which has traditionally made it difficult to isolate and evaluate their individual effects on the material's properties.^[^ ^30^, ^41^ ^]^ Despite these different types of defects, both MOFs feature identical catalytic sites with the same number of open metal sites per Zr_6_ cluster under optimal conditions, thereby maintaining equivalent intrinsic activity. Nevertheless, the distinct defect types result in different catalytic site densities. Surprisingly, UiO‐66L, which possesses a higher density of catalytic sites, demonstrated lower activity across multiple organic catalytic reactions compared to UiO‐66C with a lower catalytic site density. This suggests that an increase in the density of catalytic sites does not necessarily lead to enhanced performance. Instead, an optimal density exists, beyond which further increases in density lead to a decline in catalytic activity, forming a volcano‐shaped relationship. A combination of diffusion studies using ^129^Xe pulsed field gradient (PFG) nuclear magnetic resonance (NMR) and density functional theory (DFT) calculations revealed that UiO‐66L, despite having larger mass transport channels, exhibited slower diffusion compared to defect‐free UiO‐66 (UiO‐66I), resulting in reduced activity. This is attributed to the open metal sites serving as both catalysis and adsorption sites under high site densities, leading to strong guest molecule interactions in confined environments and increased diffusion resistance. The self‐adsorption of substrates at catalytic sites introduces intrinsic diffusion resistance, preventing further enhancement of activity. Compared to UiO‐66I, UiO‐66C features significantly larger transport channels; however, adsorption of guest molecules at its catalytic sites leads to a comparable diffusion rate. Nevertheless, the higher intrinsic activity of the open metal sites in UiO‐66C results in enhanced overall catalytic performance. This inherent self‐limiting diffusion behavior underscores the trade‐off between intrinsic activity and catalytic site density, establishing an upper limit for catalytic performance.

## 2 Results and Discussion

### 2.1 Controlling Catalytic Site Densities Through the Introduction of Exclusive Linker and Cluster Defects

To modulate catalytic site densities, we introduced distinct types of defects, namely cluster defects and linker defects, into UiO‐66 (Figure 2a). The removal of the organic linkers from the UiO‐66 structure results in the formation of linker defects (UiO‐66L), leaving open metal sites on the two adjacent metal clusters. On the other hand, cluster defects UiO‐66 (UiO‐66C) can be formed upon the removal of the [Zr_6_O_4_(OH)_4_]^12+^ cluster and the surrounding 12 organic linkers, resulting in the creation of an open metal site on the adjacent 12 clusters. To maintain the same intrinsic activity of the Zr_6_ clusters, which serve as catalytic sites, the number of missing linkers per Zr_6_ cluster is kept constant and contains four defect sites per cluster. As a result, the two samples, despite having the same catalytic Zr_6_ clusters, differ in their catalytic site densities due to the different defect types (Figure 2b). UiO‐66C, which lacks some of the metal clusters, has a lower catalytic site density. Additionally, a defect‐free UiO‐66 (UiO‐66I) sample was synthesized under optimized conditions to serve as a standard reference.

UiO‐66C was synthesized by introducing Zn as coordination competitors and templates, followed by acid etching to selectively remove Zn clusters (Figure S1, Supporting Information). Under defect‐free UiO‐66 conditions, this approach minimized the formation of linker defects, resulting in a *reo* 8‐connectivity topology with nearly ordered cluster defects (Zn: Zr = 1:1, Table S1; Figures S2–S4, Supporting Information). Powder X‐ray diffraction (PXRD) confirmed characteristic *reo* diffraction peaks around 4‐7°, and thermogravimetric analysis (TGA) showed a BDC to Zr_6_ ratio of 4:1, consistent with the formula [Zr_6_O_4_(OH)_4_(BDC)_4_], revealing four open metal sites per cluster (Figure 2a,b). For the synthesis of UiO‐66L, a mixed‐linker approach was used, with terephthalic acid (H_2_BDC) partially substituted by trans‐1,4‐cyclohexanedicarboxylic acid (H_2_CDC), followed by selective CDC removal at 325 °C, yielding UiO‐66L with four missing linkers per cluster under an optimized CDC‐to‐BDC ratio of 6:4 (Figure 2b; Tables S2 and S3; Figures S4–S11, Supporting Information). PXRD and NMR confirmed structural integrity and complete CDC removal (Figure 2a; Figures S12–S14, Supporting Information), while acid‐base titration verified similar open metal sites in both UiO‐66L and UiO‐66C (Figures S15 and S16; Table S4, Supporting Information). Moreover, the scanning electron microscopy (SEM) and transmission electron microscopy (TEM) showed UiO‐66 with highly intergrown cuboctahedral morphology, whereas UiO‐66C had smaller particles. Elemental mapping verified homogeneous Zr, C and O distribution in the framework (Figures S17 and S18, Supporting Information).

**Figure 3:**
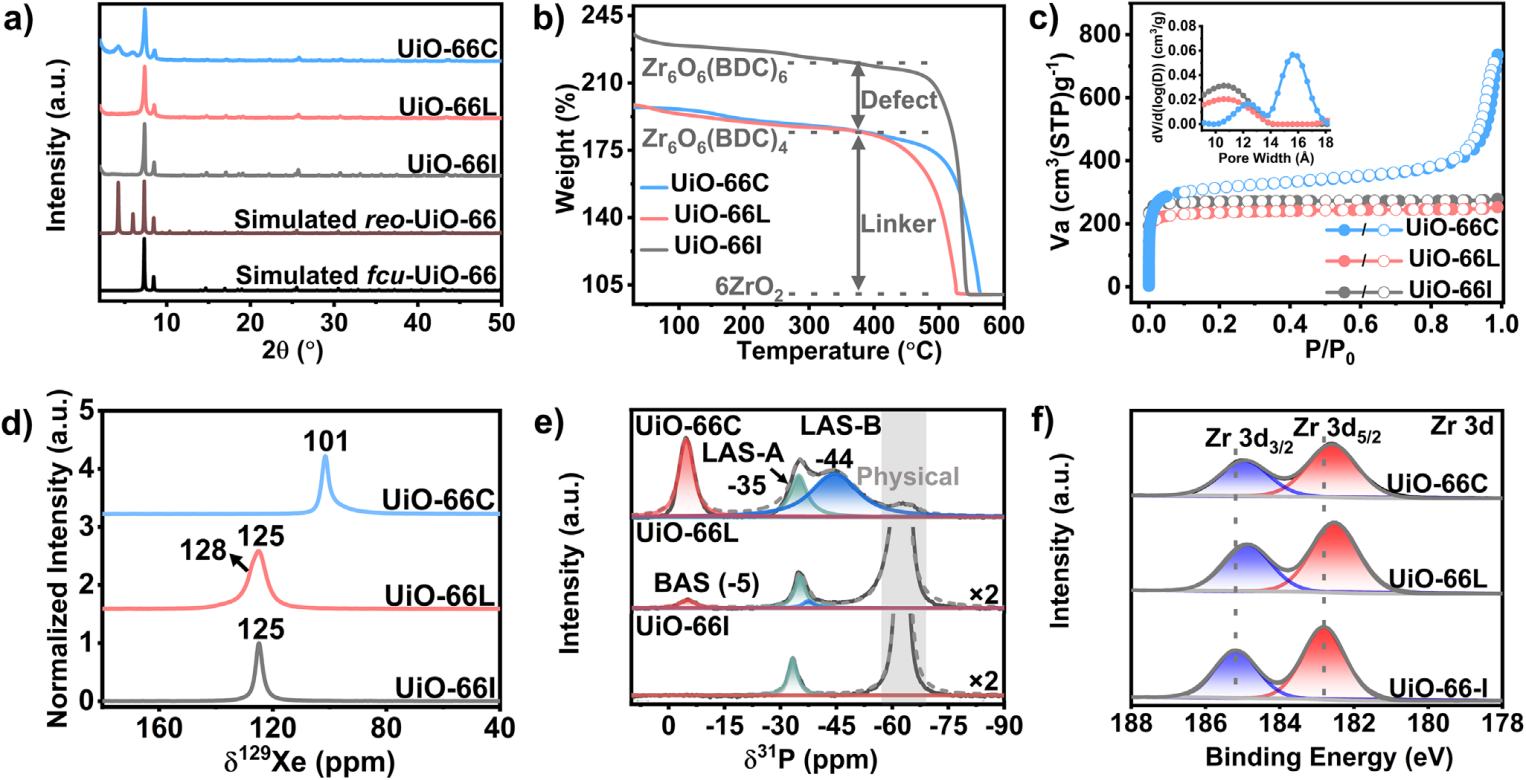
a) PXRD pattern, b) TGA curves, c) N_2_ isotherm and pore size distribution, d) ^129^Xe MAS NMR spectra, e) ^31^P MAS NMR spectra of TMP adsorbed, f) XPS Zr 3d spectra of UiO‐66‐I, UiO‐66L, and UiO‐66C.

To confirm exclusive defects, pore changes due to defect types were analyzed using N_2_ adsorption‐desorption isotherms (**Figure** **3**c), ^129^Xe magic angle spinning (MAS) NMR (Figure 3d), and ^31^P MAS NMR with trimethylphosphine (TMP) as a probe molecule (Figure 3e; Figure S19, Supporting Information). As anticipated, UiO‐66C showed increased specific surface area and additional 1.6 nm pores compared to UiO‐66L and UiO‐66I attributed to cluster defects, along with enhanced pore volume (Figure S20 and Table S5, Supporting Information). The hysteresis loop observed in UiO‐66C at relatively high pressure is attributed to the particle packing effect resulting from its small particle size (Figures S17 and S18, Supporting Information).^[^ ^42^ ^] 129^Xe NMR confirmed pore distribution by observing chemical shifts affected by interactions with pore walls.^[^ ^43^, ^44^ ^]^ The chemical shift value in ^129^Xe correlates with pore size, where a smaller chemical shift indicates a larger pore size. UiO‐66C exhibited a peak at 101 ppm (versus 125 ppm for UiO‐66I), consistent with its larger pores (Figure S21, Supporting Information). In contrast, UiO‐66L retained a 125 ppm peak, suggesting a pore size comparable to UiO‐66I, as confirmed by similar N_2_ adsorption isotherms.^[^ ^45^ ^]^ Shoulder peaks at around 128 ppm in UiO‐66L indicates the interaction of ^129^Xe molecules with defects (further confirmed through ^129^Xe PFG NMR, vide infra).^[^ ^44^ ^]^ The δ^31^P shift with TMP adsorption indicates Brønsted acid sites at ‐5 ppm, attributed to µ_3_‐OH and Zr‐OH capping groups,^[^ ^46^, ^47^, ^48^ ^]^ with UiO‐66C showing greater TMP adsorption and faster desorption at ‐63 ppm due to larger pores. Lewis acid sites (LAS) at ‐35 ppm (LAS‐A) were assigned to the surface Lewis acid sites located outside the MOFs particles, while the LAS‐B sites, observed at ≈‐44 ppm, were assigned to Lewis acid sites on internal defects. The TMP adsorption in UiO‐66C indicates higher internal defects accessibility due to larger pores, whereas UiO‐66L showed limited TMP adsorption on partially accessible open metal sites.

X‐ray photoelectron spectroscopy (XPS) was further employed to investigate chemical composition changes due to different defect types (Figure S22, Supporting Information). In the O 1s spectrum, the peak percentages of C‐O‐Zr exhibited a decline in both UiO‐66L and UiO‐66C, while the relative ratios of Zr‐O‐Zr and Zr‐O‐H to C‐O‐Zr increased compared to UiO‐66I, confirming the defect generation. Notably, similar peak percentages suggest comparable defect quantities in both UiO‐66L and UiO‐66C. In the Zr 3d spectra, the peaks at 185.2 and 182.8 eV showed Zr‐O bond formation, with lower binding energies for UiO‐66L and UiO‐66C due to increased Zr electron density and linker absence per cluster (Figure 3f). The absence of changes in the µ_3_‐O vibration bands ≈ 664 cm^−1^ in the fourier transform infrared spectra confirms that the clusters in the two defective samples remain undeformed, owing to the relative orderly distribution of defects (Figure S23, Supporting Information).^[^ ^49^ ^]^ X‐ray absorption spectroscopy at the Zr K‐edge (18022 eV, Figure S24, Supporting Information) demonstrated structural consistency across samples. Extended X‐ray absorption fine structure (EXAFS) revealed minimal changes in Zr‐O distances but reduced Zr‐Zr intensity in UiO‐66L and UiO‐66C due to broader distribution of between Zr atoms, particularly in UiO‐66C, aligning with expected defect impacts while preserving structural integrity. These changes are in line with expectations, indicating that despite the introduction of different defects, the overall structural integrity of the nodes remains preserved.

To more directly visualize the defects, integrated differential phase contrast scanning transmission electron microscopy was employed. The UiO‐66I exhibit well‐defined lattice arrangements along the (112) plane, closely matching the theoretical structural model (highlighted by yellow boxes, Figure S25a, Supporting Information). In contrast, the UiO‐66L sample clearly shows structural features indicative of missing linkers (highlighted by red boxes). Comparative analysis with the theoretical UiO‐66I model reveals the absence of horizontally aligned BDC linkers in UiO‐66L (Figure S25, Supporting Informationb). Additionally, the measured lattice spacing of 1.51 nm is in excellent agreement with the theoretical value of 1.47 nm, providing direct structural evidence for the presence of linker defects. Unfortunately, although lattice fringes were observed in UiO‐66C, no defect‐related features could be identified (Figure S26, Supporting Information).

### 2.2 The Relationship Between Catalytic Site Density and Catalysis

**Figure 4:**
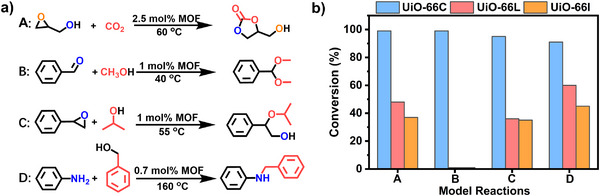
a) Model catalytic reactions (A: the cycloaddition reaction of CO_2_, B: acetalization of aldehydes, C: ring‐opening reaction of styrene oxide, D: N‐alkylation), b) conversion of four model reactions of UiO‐66C, UiO‐66L, and UiO‐66I.

To assess the impact of catalytic site density on catalysis, model reactions were studied, including those involving Zr sites as Lewis acids or Brønsted acids, as well as hydrogen transfer reactions. Specifically, we studied the cycloaddition reaction of CO_2_, ring‐opening reaction of styrene oxide, acetalization of aldehydes, and N‐alkylation (**Figure** **4**a; Figures S27–S35, Supporting Information). Among the three samples, UiO‐66C consistently showed the highest activity in these four reactions, outperforming both UiO‐66L and UiO‐66I. Moreover, UiO‐66L exhibited catalytic activity comparable to or only marginally better than UiO‐66I (Figure 4b; Tables S6–S8, Supporting Information). Although UiO‐66L features defect‐induced open metal sites and enlarged transport channels, its catalytic activity is comparable to that of the defect‐free UiO‐66I. Moreover, this unexpected outcome was particularly evident when comparing UiO‐66C (low catalytic site density) and UiO‐66L (high catalytic site density). Instead of a linear increase, the catalytic activity declined with increasing site density beyond a critical threshold.

### 2.3 Mechanism

**Figure 5:**
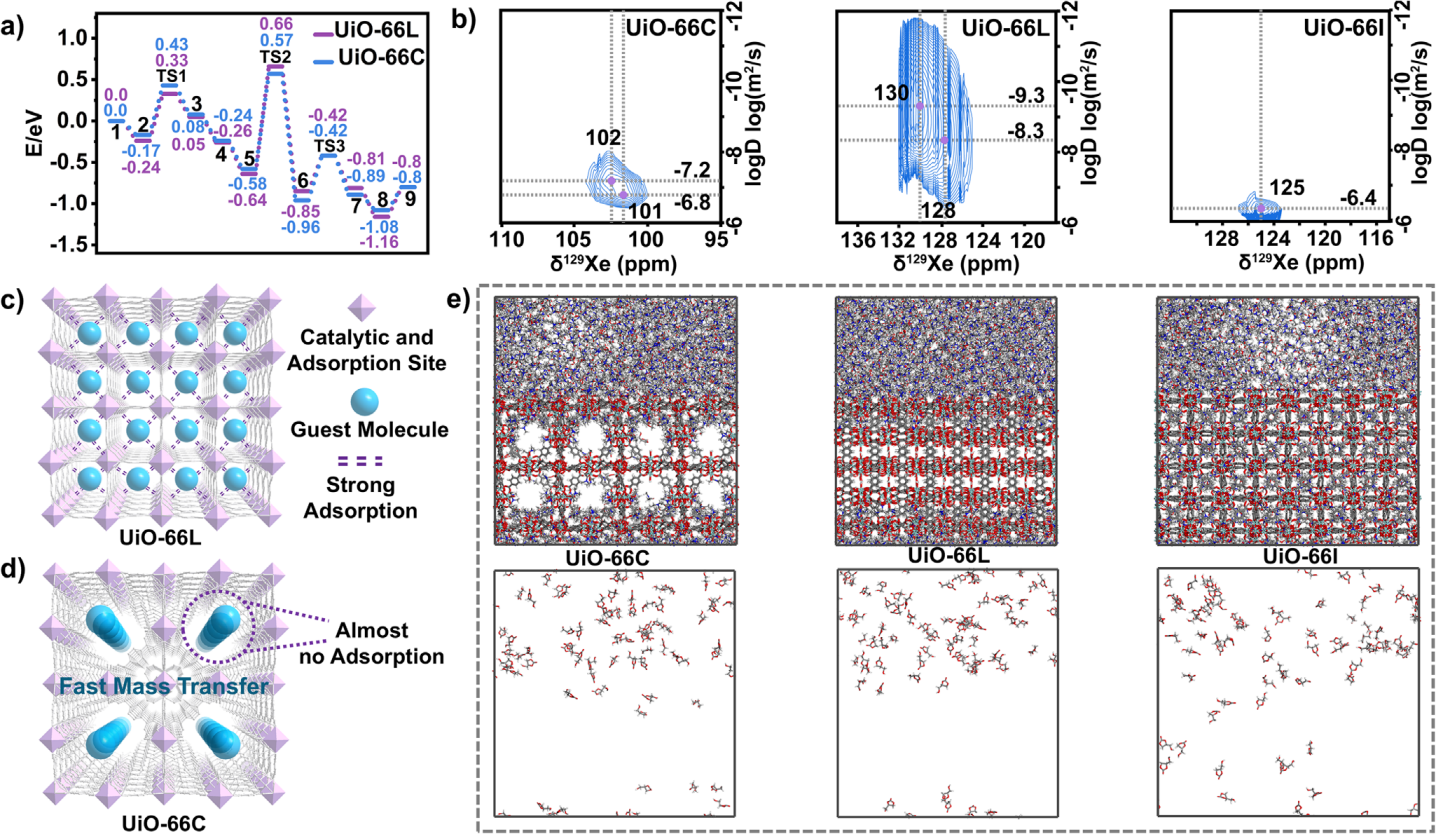
a) Calculated energy profiles for the cycloaddition reaction of CO_2_ over the UiO‐66C and UiO‐66L catalysts, b) the DOSY spectra of UiO‐66C, UiO‐66L and UiO‐66I, the illustration of the mass transfer rates of c) UiO‐66L and d) UiO‐66C, e) distribution of CO_2_ cycloaddition products and DMA in UiO‐66C, UiO‐66L and UiO‐66I after 5 ns MD simulations, with the distribution of products (removal of MOFs and DMA) shown below.

In order to gain insight into the reason behind the low catalytic activity observed in UiO‐66L with a higher density of catalytic sites, it is essential to consider the intrinsic activity and diffusion effects. The presence of four open metal sites per cluster in both UiO‐66L and UiO‐66C ensures that the catalytic sites remain identical across both samples despite the introduction of two distinct types of defects. As a result, their intrinsic activity should remain comparable. To validate this, the CO_2_ cycloaddition reaction was selected for further investigation using DFT. The catalytic mechanism of this reaction was studied, and it was found that the rate‐determining step (TS2) of the CO_2_ cycloaddition reaction is the epoxide ring‐opening (Figure S36, Supporing Information). The energy barrier associated with this step on UiO‐66L (0.66 eV) is comparable to that for UiO‐66C (0.57 eV) (Figure 5a; Figure S37, Supporing Information). This indicates that the intrinsic activity of the two samples is nearly identical, and the observed difference in catalytic activity must be attributed to other limiting factors.

Diffusion was evaluated through a series of ^129^Xe PFG NMR experiments using a simulated‐echo pulse sequence (Figure S38, Supporting Information). The diffusion ordered spectroscopy (DOSY) spectra show that the effective diffusion coefficients of the xenon molecules adsorbed on active sites (δ^129^Xe = 128 and 130 ppm) in UiO‐66L were significantly lower than those observed in UiO‐66C. The diffusion in UiO‐66C (δ^129^Xe = 101 and 102 ppm) was slightly lower than that in UiO‐66I (**Figure** **5**b). The introduction of defects in UiO‐66I was expected to increase mass transport channels and, consequently, enhance diffusion. However, the diffusion rate in UiO‐66L was unexpectedly lower than anticipated, despite having larger transport windows between the pores compared to UiO‐66I. Furthermore, the diffusion in UiO‐66C was slower than in UiO‐66I, even though it possessed transport windows three times larger. This unexpected behavior can be attributed to the fact that the defect‐generated open metal sites not only function as catalytic centers but also exhibit strong adsorption interactions with guest molecules when the pore size is comparable to that of the guest species (Figure 5c). While the open metal sites in UiO‐66C adsorb guest molecules and increase diffusion resistance, the enlarged pore size offsets this effect, minimizing the impact on overall diffusion (Figure 5d). In contrast, the interactions between open metal sites and guest molecules in UiO‐66L lead to a more pronounced reduction in diffusion, even with larger mass transport windows.

To further validate these findings, molecular dynamics (MD) simulations were performed to gain insight into the diffusion rates of molecules in the UiO‐66C, UiO‐66L, and UiO‐66I frameworks. The CO_2_ cycloaddition product molecules (glycerol‐1,2‐carbonate) and solvent molecules N, N‐dimethylacetamide (DMA) were observed to enter the empty pores of the UiO‐66I, UiO‐66C, and UiO‐66L frameworks at varying rates, contingent on the distinct porosities (Figure 5e; Figure S39, Supporting Information). The calculated diffusion coefficients for the product molecules in UiO‐66I, UiO‐66L, and UiO‐66C are 6.9, 4.5 and 5.5 (10^−6^ cm^2^ s^−1^), respectively (Figure S40, Supporting Information). It is noteworthy that in the case of UiO‐66C, the number of molecules observed to enter the empty pores were significantly greater. In contrast, only trace amounts of the product and solvent molecules were observed to enter the UiO‐66L framework, while the original empty pores of UiO‐66I were almost entirely filled with the product and solvent molecules. The MD trajectories demonstrate that product molecules can adsorb at the open Zr sites in both UiO‐66C and UiO‐66L, which impedes the entry of subsequent molecules (Figures S41 and S42, Supporting Information). The higher diffusion rate of the molecules in UiO‐66C than that in UiO‐66L can be attributed to the larger pores resulting from the absence of clusters, which allows the molecules to enter the MOFs channels with greater ease. In contrast, UiO‐66I, which lacks interaction sites, showed a higher diffusion rate than UiO‐66L, likely due to the absence of adsorption effects on the guest molecule. These results are consistent with the ^129^Xe PFG NMR diffusion experiments, further confirming that the adsorption of guest molecules at defect‐induced open metal sites increases diffusion resistance, even with larger mass transport channels.

Both computational and experimental results indicate that the low activity of UiO‐66L with high catalytic site density, compared with UiO‐66C, can be attributed to self‐adsorption at catalytic sites, leading to diffusion resistance becoming the limiting factor. In this case, increasing the density of catalytic sites can be ineffective for enhancing reaction performance; instead, it leads to a reduction in activity. As the density of catalytic sites increases, the transport channels within the framework become narrow. When the size of these channels approaches that of the guest molecule, the interaction between the catalytic sites and the guest molecule intensifies, which has a detrimental impact on guest molecule diffusion and ultimately reduces the reaction activity.

Despite both UiO‐66L and UiO‐66I having the same density of clusters, the intrinsic activity of defect sites in UiO‐66L is higher than that of saturated metal centers in UiO‐66I. Therefore, one would expect UiO‐66L will exhibit higher catalytic activity than UiO‐66I. However, their similar catalytic performance seems counterintuitive. This discrepancy is likely due to the strength of the interactions between the catalytic sites and guest molecules. The open metal sites in UiO‐66L, while possessing higher intrinsic catalytic activity, also exhibit stronger adsorption interactions with guest molecules. At high catalytic site densities, especially when the guest molecule size approaches the pore size, these adsorption interactions increase diffusion resistance. This increased diffusion resistance offsets the increasing from the enhanced intrinsic activity of the defect sites, resulting in overall catalytic performance similar to that of UiO‐66I, where diffusion is less restricted.

It can be concluded from this study that while increasing catalytic site density can enhance the catalytic activity, there is an upper limit beyond which further increases lead to diminished performance. From an atomic‐level perspective, when catalytic sites are densely packed, it is crucial to ensure sufficient mass transfer channels for diffusion. As the size of the transport channel approaches that of guest molecules, the catalytic sites no longer solely function as catalytic sites but rather act as adsorption sites, significantly impacting the diffusion of guest molecules. This critical density is not determined by the transport channel size that merely allows guest molecules to enter, nor by the size where the intrinsic diffusion rate of the channel matches the reaction rate. Instead, the optimal transport channel size must account for the adsorption interactions between highly dispersed, uniformly distributed catalytic sites and guest molecules. These interactions influence the diffusion rate of guest molecules, which must be balanced with the reaction conversion rate. This balance ultimately defines the maximum catalytic site density for achieving optimal performance.

When considering the limit of catalytic site density, the space between adjacent sites serves as the diffusion channel, thereby determining the diffusion of reactant molecules. During catalysis, chemical adsorption between the catalytic sites and the guest molecule is necessary for the reaction to proceed, and usually occurs after physical adsorption. As a result, in a restricted transport channel, diffusional resistance is unavoidable due to the strong interaction between the catalytic sites and the guest molecules. It is therefore essential to consider the size of the guest molecules when optimizing the density of catalytic sites, in order to match the host catalyst's mass transfer channels from both thermodynamic and kinetic standpoints. For specific reactions, catalysts with higher intrinsic activity and strong adsorption require larger transport channels to achieve high diffusion rates, thereby reducing the need for a high density of catalytic sites. On the other hand, catalysts with lower intrinsic activity and weaker adsorption require a higher density of catalytic sites to achieve optimal catalytic performance. The optimal catalytic performance is achieved when the intrinsic reaction rate is well‐matched with the mass transfer rate. Therefore, there exists a trade‐off between the intrinsic activity and the catalytic site density, as the catalytic centers also serve as adsorption sites that obtain self‐limiting diffusion.

## 3 Conclusion

The controlled synthesis of UiO‐66 with a single type of defect enabled the production of materials with different catalytic site densities. Investigating multiple organic catalytic reactions revealed that UiO‐66L, despite having a higher density of catalytic sites, exhibited lower specific activity. This indicates the existence of an upper limit to catalytic site density. As the density approaches this limit, catalytic sites function not only as active sites but also as adsorption sites for guest molecules, introducing intrinsic diffusion resistance that restricts further activity enhancement. This work elucidates the contribution of defect types to catalysis and highlights the trade‐off between intrinsic activity and catalytic site density. This fundamental balance extends beyond MOFs and must be considered in the rational design of any heterogeneous catalyst. As catalysis research enters an era of rational design—particularly with the rapid advancement of single‐atom catalysts—researchers are now able to precisely control the spatial distribution of active sites and fine‐tune their optimal density. This enables enhanced space‐time yields, bringing catalytic performance closer to theoretical limits and meeting the demands of industrial‐scale production.

## 4 Experimental Section

### Synthesis of UiO‐66I

UiO‐66I was synthesized using an optimized approach based on a previously established method.^[^ ^45^ ^]^ The synthesis mixture was created by adding 470 mg of ZrCl_4_ (2.02 mmol), 0.36 mL of 37% HCl (4.04 mmol), and 670 mg of terephthalic acid (H_2_BDC) (4.04 mmol) sequentially to a 25 mL Teflon‐lined autoclave filled with 12 mL of N, N’‐dimethylformamide (DMF). Once the reagents were fully dissolved, the mixture was placed in an oven that had been preheated to 220 °C. After a reaction time of 24 h, the autoclave was taken out and quickly cooled by submerging it in a bucket of cold tap water. When the autoclave had cooled to room temperature, the resulting white solids were collected through filtration and thoroughly washed with DMF and methanol. The washed product was then dried overnight in an oven at 60 °C.

### Synthesis of UiO‐66L

The concept of a single linker defect in UiO‐66 was first introduced by De Vos and colleagues.^[^ ^50^ ^]^ In this study, the synthesis conditions were optimized to minimize the occurrence of inherent defects. To achieve this, 470 mg of ZrCl_4_ (2.02 mmol) was dissolved in 12 mL of N, N‐dimethylformamide within a 25 mL Teflon‐lined autoclave, followed by the addition of varying proportions of H_2_BDC and trans‐1,4‐cyclohexanedicarboxylic acid (H_2_CDC) as outlined in Table S2 (Supporting Information). After all reagents had dissolved, 0.36 mL of 37% HCl (4.04 mmol) was added. The resulting solution was then allowed to react at 220 °C in a conventional synthesis oven for 24 h. Following the reaction, the autoclave was taken out of the oven and quickly cooled by immersing it in a bucket of cold tap water. The resulting white solids were collected through filtration and thoroughly washed with DMF and methanol. Subsequently, the mixed‐linker UiO‐66 with varying BDC and CDC ratios was heated in air at 325 °C for 2 h. Finally, the solids were soaked in methanol for 24 h and then dried under vacuum at 65 °C. The sample with a mixed‐linker BDC/CDC ratio of 4:6 was designated UiO‐66L. The synthesis methods for UiO‐66‐1, UiO‐66‐2, UiO‐66‐3, UiO‐66‐4, UiO‐66‐5, UiO‐66‐7, UiO‐66‐8, and UiO‐66‐9 were identical to that of UiO‐66L, with their corresponding H_2_BDC/H_2_CDC ratios provided in Table S2 (Supporting Information).

### Synthesis of UiO‐66C

The bimetallic (Zn, Zr)‐UiO‐66 was synthesized using a method previously reported, with further optimizations implemented.^[^ ^51^ ^]^ In this process, 470 mg of ZrCl_4_ (2.02 mmol) and of Zn(NO_3_)_2_·6H_2_O (2.02 mmol) were dissolved in 12 mL of N,N‐dimethyl formamide. Following this, 670 mg of H_2_BDC (4.04 mmol) and 0.36 mL of 37% HCl (4.04 mmol) were added to the solution. The mixture was sonicated for 30 min before being transferred to a Teflon‐lined autoclave, where it was heated to 220 °C for 24 h. After this period, the autoclave was taken out of the oven and quickly cooled by immersing it in a bucket of cold tap water. Once the autoclave had cooled to room temperature, the resulting white solids were filtered out and thoroughly washed with DMF and methanol. In the next step, the bimetallic UiO‐66 with varying ratios of Zn and Zr was stirred in HCl (pH 1) for 10 min, then filtered and washed with water until reaching a neutral pH, followed by a wash with methanol. The solids were subsequently soaked in methanol for 24 h and dried under vacuum at 65 °C. The synthesis methods for UiO‐66‐10, UiO‐66‐11 and UiO‐66‐12 were identical to that of UiO‐66C, with their corresponding ZrCl_4_/Zn(NO_3_)_2_·6H_2_O ratios provided in Table S1 (Supporting Information).

## Conflict of Interest

The authors declare no conflict of interest.

## Supporting information



Supporting Information

## Data Availability

The data that support the findings of this study are available from the corresponding author upon reasonable request.
