# Supplementary material for: Beyond Density: Unveiling the Trade‐Off in Catalytic Site Optimization Using Defect‐Engineered UiO‐66
Source: Adv Sci (Weinh). 2025 Sep 2;12(43):e10578. doi: 10.1002/advs.202510578 (PMC12631877; doi:10.1002/advs.202510578)
Supplement: Supplementary file 1 — Supporting Information [file ADVS-12-e10578-s001.docx]

Supporting Information

Beyond Density: Unveiling the Trade-off in Catalytic Site Optimization Using Defect-Engineered UiO-66

Guo-Ying Han^1^†, Yi Ji^2^†, Xiang-Yu Li^3^†, Yu Gai^1^, Guo-Zhen Hou^1^, Qin-Yi Cheng^1^, Zhong Zhang^1^, Yiwei Liu^1^, Jia Wang^3^, Pascal Van Der Voort^4^, Guangjin Hou^2^*, Xin-Ping Wu^3^*, Gaohong He^5^*, Xiao Feng^1^*

**Methods**

**General considerations**

All chemicals were sourced from Energy Chemical, Bidepharm, Aladdin, or DaMao and were used as received without additional purification. Powder X-ray diffraction (PXRD) data were obtained using a Bruker D8 Advance X-ray diffractometer with Cu Kα radiation, covering a 2θ range of 2-50° at a scanning speed of 10°/min. Additionally, ^1^H and ^13^C liquid nuclear magnetic resonance (NMR) spectra were recorded on a Bruker AV-400 spectrometer. Thermogravimetric analysis (TGA) profiles were recorded over a temperature range of 30-600 °C using a PerkinElmer Simultaneous Thermal Analyzer (STA) 6000, with a heating rate of 5°/min in air flow. Nitrogen adsorption-desorption isotherms were determined at -196 °C using a Micromeritics 3Flex instrument. Prior to gas adsorption measurements, all samples were degassed at 120 °C under vacuum for 12 hours. The total surface area was determined using the BET equation. Fourier transform infrared spectroscopy (FTIR) spectra were obtained with a Thermo Scientific Nicolet 6700. X-ray photoelectron spectroscopy (XPS) data were collected using a Thermo Fisher Scientific K-Alpha X-ray photoelectron spectrometer. Scanning electron microscopy (SEM) images, along with the corresponding energy-dispersive X-ray spectrometry (EDS) mapping, were acquired using a JEOL JSM-7900F Ultrahigh Resolution Field Emission Scanning Electron Microscope. Transmission electron microscopy (TEM) images and elemental analysis were conducted using a JEM-F200 Field Emission Transmission Electron Microscope. The high-angle annular dark-field scanning transmission electron microscopy (HAADF-STEM) and integrated differential phase contrast STEM (iDPC-STEM) images were acquired using a Thermo Fisher Dual Spherical Aberration Corrected Transmission Electron Microscope. The accelerating voltage is 300 kV, the convergence semi-angle is 5.0 mrad. The electron beam current is 2.0 pA, and the collection angles were set as 5 ~ 19 mrad. The potentiometric acid-base titration curves were generated using the Hogon CT-1Plus multifunctional automatic titrator, with calibration conducted using commercial pH buffers of 4.00, 6.86, and 9.18. The conversion of these model reactions was determined by integrating the peaks from an Agilent 8860 gas chromatography system paired with an Agilent 5977B mass spectrometer. Gas chromatography-mass spectrometry (GC-MS) analysis was performed on an Agilent 8860-5977B GC/MSD system equipped with a capillary column (HP-5MS, Agilent Technologies, 30 m length, 0.25 mm i.d., 0.25 μm film thickness). X-ray absorption fine structure (XAFS) spectra at the Zr K-edge (17998 eV) were recorded at the BL14W1 station of the Shanghai Synchrotron Radiation Facility (SSRF) in China. The XAFS data were obtained under ambient conditions in transmission mode with a Lytle detector. Each sample was pelletized into an 8 mm diameter disk, composed of 20 mg of sample mixed with 80 mg of graphite, and the extended X-ray absorption fine structure XAFS spectra were recorded in fluorescence mode at room temperature. The raw data were energy-calibrated using Zr foil, then underwent background subtraction, normalization, and Fourier transformation according to standard procedures with the ATHENA program.^[1]^ The Zr K-edge E_0_, approximately 17998 eV, was determined from the highest peak of the first derivative of the X-ray absorption near-edge spectroscopy (XANES). Following this, the k³-weighted χ(k) data in k-space were Fourier transformed into real R-space after applying a Hanning window function (Δk = 1.0 Å⁻¹).

**Solid-state NMR measurements**

All solid-state NMR experiments were conducted on a Bruker Avance III spectrometer at 14.1 T (^1^H Larmor frequency at 600.13 MHz).

^1^H and ^13^C magic angle spinning (MAS) NMR experiments were performed using a 3.2 mm HXY probe with a MAS rate of 20 kHz. The samples were dehydrated before NMR measurements by vacuum annealing at 120 ℃ and < 10^-3^ Pa overnight. ^1^H Hahn-echo spectra were recorded with an echo delay of 50 μs, a recycle delay of 10.5 s, and 16 scans. ^1^H→^13^C cross polarization (CP) spectra were recorded with a CP contact time of 3 ms, a ramped (90 %-110 %) RF field on ^1^H, ^1^H SPINAL-64 decoupling with *γB*_1_/2π = 67 kHz, a recycle delay of 2.7 s, and 4096 scans. ^1^H chemical shifts are referenced to adamantane (1.74 ppm). ^13^C chemical shifts are referenced to adamantane (CH) at 29.45 ppm.

^31^P MAS NMR experiments were performed using a 3.2 mm HXY probe with a MAS rate of 20 kHz. ^31^P direct-excitation spectra were recorded with a π/4 pulse of 3.3 μs, ^1^H SPINAL-64 decoupling with *γB*_1_/2π = 67 kHz, a recycle delay of 15 s, and 512 scans. ^31^P chemical shifts are referenced to (NH_4_)_2_HPO_4_ at 1.13 ppm. Trimethylphosphine (TMP) adsorption experiments were carried out in the following steps. The dehydrated UiO-66 samples were firstly exposed to excessive TMP and balanced at 60 ℃ for 1 hour, followed by evacuation at 60 ℃ for 1 hour.

^129^Xe NMR experiments were performed using a Diff-50 diffusion probe with a 5 mm NMR coil insert and a maximum magnetic field gradient strength of 1800 G/cm in the z-direction. All the measurements were conducted at 25 ℃. ^129^Xe direct-excitation spectra were recorded with a π/2 of 8 μs, a recycle delay of 10-65 s (> 5·T_1_) and 128-256 scans. ^129^Xe chemical shifts are referenced to gas-phase xenon at 0 ppm. ^129^Xe pulsed field gradient (PFG) NMR experiments were recorded using a stimulated echo (STE) sequence with a series of applied gradient strength *g* (10→300 G/m for UiO-66C and UiO-66L; 10→140 G/m for UiO-66I) in 12 steps, while the gradient duration *δ* = 0.5 ms and the diffusion time *Δ* = 5 ms were kept constant. The basic principle of PFG NMR is reported in detail elsewhere.^[2]^ It should be noted that Tanner STE sequence instead of the ones with more intervals to reduce effects of internal and background gradients, *g*_0_^[3]^, were applied in all the experiments for *T*_2_ limitation. As multiple kinds of adsorption species with varied *δ*^129^Xe may hold different diffusivity, PFG NMR results were transferred into diffusion ordered spectroscopy (DOSY) spectra^[4]^, which were processed using CONTIN mode implemented in TopSpin. The different diffusivities were estimated by direct readout in DOSY spectra. It should be stressed that the measured diffusivities should be recognized as effective diffusivities involving both intra- and inter-crystal diffusion. It requires the crystal size full filling the requirement, <*r*^2^> > 6*DΔ*, to obtain intra-crystal diffusivities. Referring to the calculated self-diffusivity for Xe in UiO-66 of 1.31×10^-9^ m^2^/s at 400 K^[5]^, it requires a crystal diameter larger than 10 μm to avoid internal Xe diffusing out of the crystal boundary, which is much larger than the samples used in this work. Moreover, the measured diffusivity in UiO-66I is ca. 4×10^-7^ m^2^/s at 298 K in this work which can be too high for a self-diffusivity one. Xenon adsorption experiments were carried out in the following steps. Dehydrated UiO-66 samples of known mass were transferred into a 5 mm outer-diameter NMR tube with a pressure valve, which were then exposed to ^129^Xe and kept at room temperature until reaching adsorption balance with gas phase at ca. 90 kPa (90.4 kPa for UiO-66C; 88.2 kPa for UiO-66L; 88.3 kPa for UiO-66I). The NMR tube were then sealed before removal from vacuum line, followed by NMR measurements. The adsorption amounts can be calculated from pressure reduction in a known-volume container (1.44 mmol/g for UiO-66C; 1.35 mmol/g for UiO-66L; 2.15 mmol/g for UiO-66I).

**Density functional theory (DFT) calculations**

The catalytic mechanism of the CO_2_ cycloaddition reaction on UiO-66L and UiO-66C were calculated using DFT. All DFT calculations were performed using the *Vienna Ab initio Simulation Package* (*VASP*) ^[6, 7]^. The Perdew–Burke–Ernzerhof (PBE) functional^[8]^ was used. The core–valence interactions were described using the projector augmented wave (PAW) method^[9, 10]^. The Brillouin zone integration was sampled using the Monkhorst-Pack scheme with a 1 × 1 × 1 *k*-point mesh, and the kinetic energy cut-off was set to 500 eV. The DFT-D3 method^[11]^ was employed to account for the long-range dispersion interactions. The effects of cavitation, electrostatics, and dispersion of the solvent N,N-Dimethylacetamide (DMA) were depicted using the implicit solvation model implemented in *VASPsol*^[12, 13]^, with a relative permittivity of ε_r_ = 37.8. The force threshold for structural optimizations was set to 0.05 eV/Å, and a convergence energy threshold of 10^-5^ eV was used for the self-consistent field calculations.

The UiO-66I structure was obtained from the Cambridge Crystallographic Data Centre (CCDC deposition number: 8895295)^[14]^. For UiO-66I, the optimized lattice parameters are a = b = c = 20.8994 Å, which is close to the experimental values (a = b = c = 20.7004 Å)^[15, 16]^. The UiO-66L structure was derived from the UiO-66I structure by the removal of eight linkers, while maintaining the connectivity of the clusters to eight linkers. The UiO-66C structure was obtained by the removal of a single cluster and its associated linkers. Each defective site (i.e., two open Zr sites) was capped with one hydroxyl group to maintain charge neutrality. The reactant molecules (CO_2_ and glycidol), the product molecule (glycidol carbonate), and the solvent molecule were all optimized within a box of 20 × 20 × 20 Å^3^. In the course of the catalytic reaction calculations, the lattice parameters were fixed. The constrained optimization scheme^[17]^ and the CI-NEB^[18]^ method were used for the purpose of searching transition state structures.

**Molecular dynamics (MD) simulations**

Diffusion rates of glycerol-1,2-carbonate and solvent molecules (DMA) were calculated based on the MD simulations performed using *GROMACS 2024*^[19]^ with the Universal Force Field (UFF)^[20]^. In the simulations, periodic boundary conditions were applied in all directions. The systems were initially minimized in terms of energy using the steepest descent method, followed by a MD simulation with the NPT ensemble at 333 K and 1 atm for 500 ps of equilibrium. Subsequently, a MD simulation was conducted utilizing the NVT ensemble at 333 K for a 5 ns production run. This was done to ensure that the simulation time was sufficiently long and that the products and DMA were still diffusing toward the middle region of the frameworks. The temperature and pressure were controlled by the V-rescale thermostat^[21]^ and the Berendsen barostat^[22]^, respectively. The long-range electrostatic interaction was computed using the particle mesh Ewald (PME) method^[23]^, with a cutoff of 1.2 nm. The partial atomic charges employed in the classical simulations were calculated using *CP2K 2022*^[24]^, the PBE functional was employed in conjunction with the DZVP-MOLOPT basis set^[25]^ and Goedecker-Teter-Hutter pseudopotentials^[26]^, with a plane-wave energy cutoff of 400 Ry. The charges of the metal-organic frameworks (MOFs) were calculated using the REPEAT method, while the charges of the product molecules and the solvent molecules were calculated using the RESP method. Similarly, the cutoff distance for the van der Waals interactions was set to 1.2 nm. Vibrations of bonds containing hydrogen atoms were constrained via the LINCS algorithm^[27]^. All MD simulations were performed with a timestep of 0.5 fs.

In the construction of the models, the 4 × 4 × 2 supercells of UiO-66I, UiO-66L, and UiO-66C were initially constructed, and subsequently, the *b* parameter of the supercells was elongated by approximately 50 Å. The dimension of the models are approximately 85 × 100.0 × 85 Å^3^, which is comparable to those reported in literature^[28, 29]^. The resulting open Zr sites on the outer surface of the MOFs structures were then capped with formic acid. *PACKMOL*^[30]^ was used to arrange molecules within specified regions of space. A total of 75 product molecules (glycerol-1,2-carbonate) and 1620 solvent molecules (DMA) were introduced into the empty space of each box^[31, 32]^. The topology files were generated using *Sobtop*^[33]^.

The diffusion rates of the product molecules were evaluated by calculating the mean square displacements (MSDs)^[34]^.

$$MSD \left( \Delta t \right)=\frac{1}{N}\sum_{i=1}^{N} <{|r_{i}(t)-r_{i}(0)|}^{2}>$$

where *N* is the number of product molecules, and $r_{i}(t)-r_{i}(0)$ represents the distance traversed by the *i*^th^ solvent molecule over a specified time interval.

**Acetalization of aldehydes**

In a typical catalytic test, 24 mg of catalyst (1 mol%, based on Zr_6_) and 77 mg of benzaldehyde (1.45 mmol) was dispersed in 1.25 mL of methanol within a 4 mL vial. Next, 56 mg of biphenyl (0.36 mmol), used as an internal standard, were added to the mixture. This solution was then magnetically stirred at 40 °C. At specified time intervals, aliquots were drawn using a syringe, filtered through a nylon filter to eliminate catalyst particles, and the products were analyzed by GC-MS.

**Ring-opening reaction of styrene oxide**

The ring-opening reactions of styrene oxide with isopropanol took place in a 4 mL vial. This involved combining styrene oxide (46 μL, 0.40 mmol), anhydrous isopropanol (1000 μL), and dodecane (90 μL, 0.40 mmol) in the vial. After the catalyst (1 mol%, calculated based on Zr_6_) was added, the vial was sealed and immersed in an oil bath set to 55 °C. Samples were collected at various time points and analyzed using GC, with results verified by GC-MS.

**Cycloaddition reaction of CO_2_**

In a typical procedure, a 4 mL vial contained 21 mg of catalyst (2.5 mol%, based on Zr_6_), 1 mL of N, N-dimethylacetamide (DMA), 37 mg of epoxide (0.5 mmol), and 38 mg of biphenyl as the internal standard (0.25 mmol). To create a pure CO_2_ atmosphere, the vial was connected to a CO_2_-filled balloon and purged multiple times with CO_2_. The catalytic cycloaddition reaction was then carried out at 60 °C. Periodically, samples of the reaction mixture were taken, mixed with ethyl acetate, and analyzed using GC-MS.

**N-alkylation reaction**

An equimolar reaction mixture consisting of aniline (470 mg, 50 mmol), benzyl alcohol (540 mg, 50 mmol), and 54 mg of catalyst (0.7 mol%, based on Zr_6_) was prepared in a vial. The vial was sealed and heated to 160 °C under solvent-free conditions. Samples taken at various reaction times were analyzed using GC-MS.

**Acid-base titrations**

Before titration, the MOFs samples were ground using a mortar and pestle. About 50 mg of the ground sample was then dispersed in approximately 60 mL of 0.01 M NaNO_3_ solution and allowed to equilibrate for 18 hours. Each titration solution was equipped with a magnetic stir bar, adjusted to a pH of 3 with 0.1 M HCl, and subsequently titrated with 0.1 M NaOH until a pH of 10.5-11 was reached. Equivalence points were identified using the first derivative of the titration curve, which plots pH against the volume of titrant added. The maximum values in this derivative curve correspond to the inflection points and indicate the equivalence points. The pKa values were calculated as the pH at half the volume of titrant required to reach the equivalence point.

**Quantitative Analysis of TGA Data**

The typical TGA curve of UiO-66 reveals three distinct stages: (1) Desolvation, where solvent molecules are removed from the porous framework, usually completed at temperatures below 150 °C; (2) Between approximately 150 and 350 °C, two structural water molecules and the compensating ligand are removed from the clusters; and (3) At temperatures above 350 °C, the organic components of the desolvated and dehydroxylated MOFs decompose, resulting in the formation of ZrO_2_. If no defects are present in UiO-66, the molecular weight ratio of six BDC linkers to ZrO_2_ should be 220.8%. However, significant variations in weight loss at temperatures above 350 °C were observed across different batches of UiO-66 in the TGA curves. In particular, most samples showed a weight loss smaller than what would be predicted by the ideal chemical formula Zr_6_O_6_(BDC)_6_ for a desolvated and dehydroxylated UiO-66. This discrepancy is due to the presence of linker defects in the framework, and the amount of missing linkers can be determined from this discrepancy.

**Quantitative Analysis of catalytic site density**

UiO-66 can exhibit two distinct types of defect: linker defects and cluster defects, both of which result in per metal cluster having four open metal sites. Here, per metal cluster is normalized as a single catalytic active site. The density of catalytic active sites per unit volume is equivalent to the density of metal clusters. Since UiO-66L does not exhibit any missing clusters, the catalytic active site density of UiO-66L is considered to be 1. Compared with UiO-66L, UiO-66C is missing 1/4 clusters, resulting in a catalytic active site density of 0.75 for UiO-66C.

**The nomenclature of the UiO-66 series samples**

To investigate the effect of initial CDC content on linker defects in UiO-66 materials, we synthesized a series of UiO-66 samples with varying linker defects, where the molar ratios of BDC to CDC were: UiO-66-1 (9:1), UiO-66-2 (8:2), UiO-66-3 (7:3), UiO-66-4 (6:4), UiO-66-5 (5:5), UiO-66L (4:6), UiO-66-7 (3:7), UiO-66-8 (2:8), and UiO-66-9 (1:9). NMR and TGA analyses revealed consistent CDC contents in the unheated samples, with the incorporated CDC amount increasing proportionally to the initial feed ratio. After thermal treatment at 325°C, UiO-66-7, UiO-66-8 and UiO-66-9 samples all underwent structural collapse, while UiO-66L maintained its crystalline integrity while exhibiting the highest linker defect, featuring four missing linkers per metal cluster.

We systematically investigated materials with different initial Zr:Zn ratios and prepared a series of defect-engineered UiO-66 materials: UiO-66-10 (Zr:Zn = 1:0.33), UiO-66-11 (Zr:Zn = 1:0.50), UiO-66-12 (Zr:Zn = 1:0.67), and UiO-66C (Zr:Zn = 1:1). With increasing initial Zn content, the defective UiO-66 samples exhibited increasingly sharper and more distinct characteristic diffraction peaks within the 4-7° range in PXRD pattern. Meanwhile, the TGA curves indicates that the the number of defects increases with higher initial zinc content.


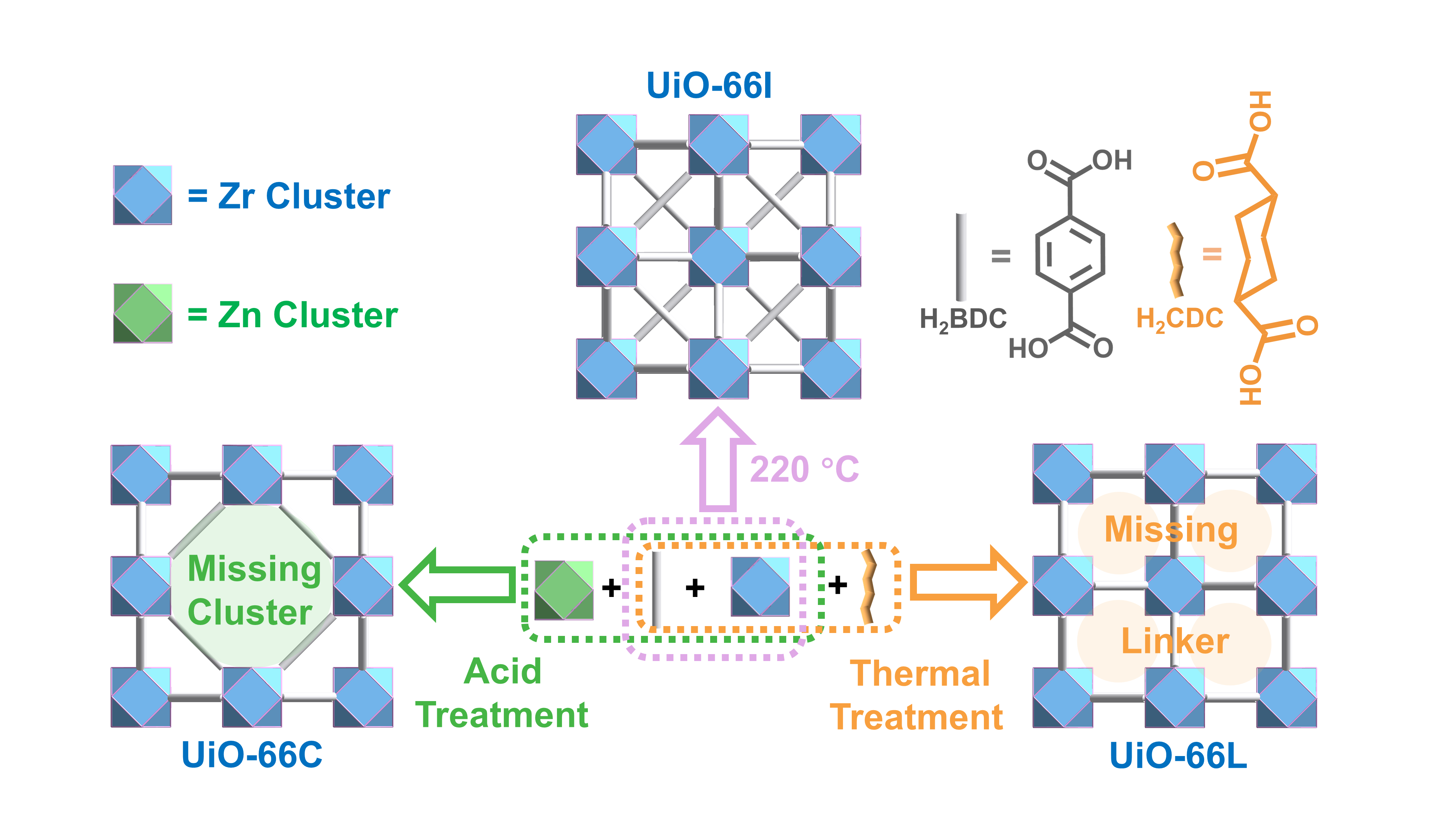


**Figure S1.** Illustrations of the synthesis of UiO-66I, followed by fabricating UiO-66C (acid treatment) and UiO-66L (thermal treatment).


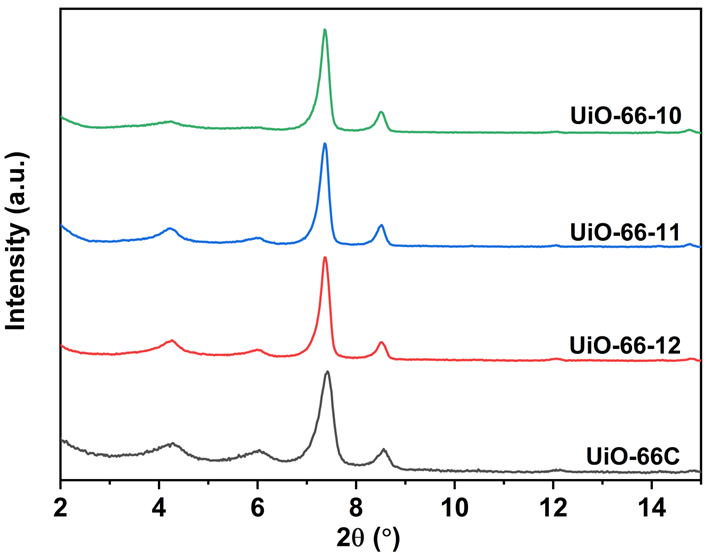


**Figure S2.** PXRD pattern of UiO-66-10, UiO-66-11, UiO-66-12, UiO-66C after treatment with 0.1 M HCl. UiO-66C observed the most distinct diffractions at approximately 4 to 6°.


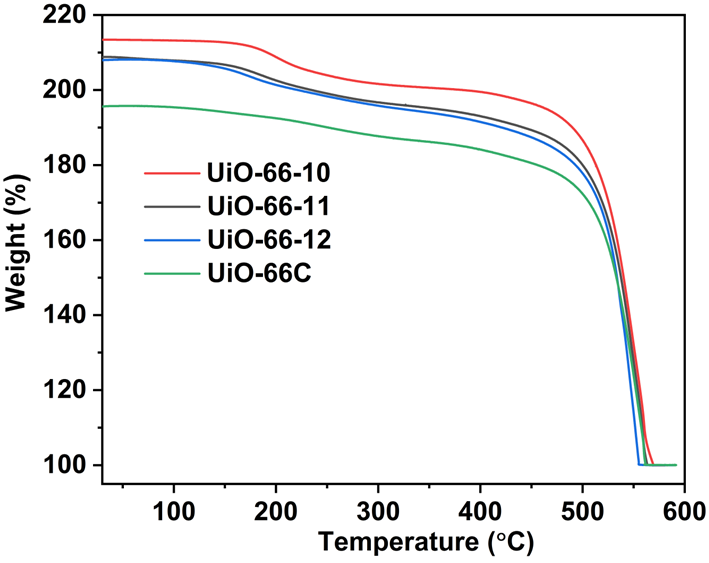


**Figure S3.** The TGA curves of UiO-66-10, UiO-66-11, UiO-66-12 and UiO-66C were shown after treatment with 0.1 M HCl. The amount of defects increased with the amount of Zn added in the synthesis process, and UiO-66C showed the largest amount of defects.


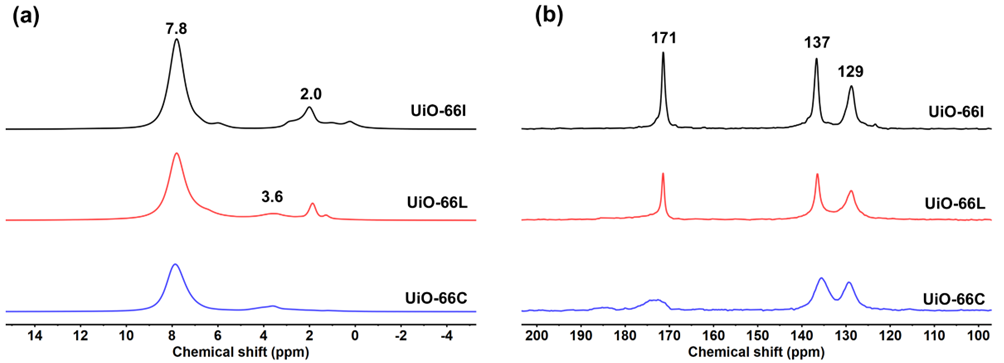


**Figure S4.** ^1^H (a) and ^13^C (b) MAS NMR spectra of UiO-66C, UiO-66L and UiO-66I.


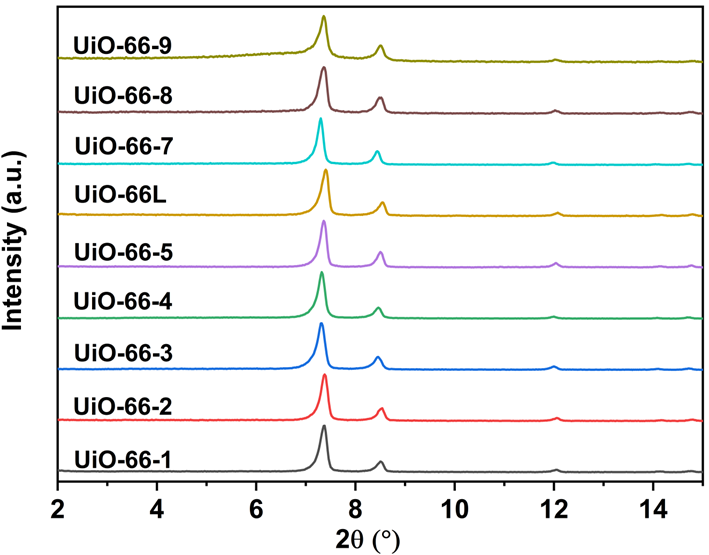


**Figure S5.** PXRD pattern of UiO-66 with different proportions of H_2_CDC before thermal decomposition of CDC.


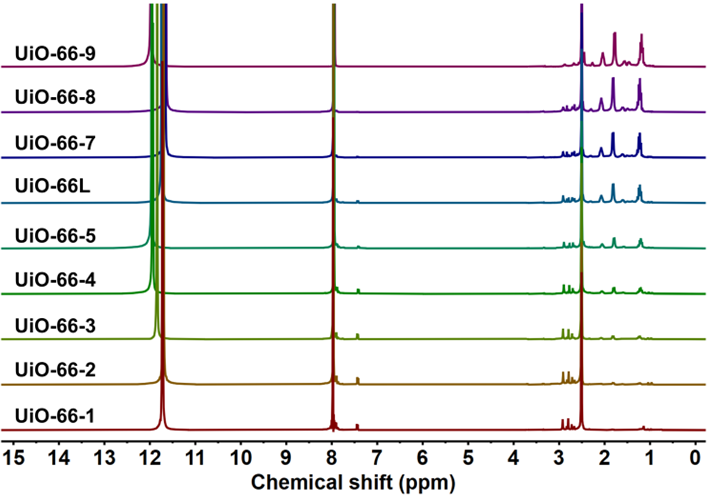


**Figure S6.** ^1^H NMR spectra of UiO-66 with different proportions of H_2_CDC after digestion in d_6_-DMSO using D_2_SO_4_. The aromatic BDC proton resonates at 8.0 ppm, while CDC gives rise to signals at 1.2 ppm, 1.8 ppm and 2.1 ppm. The resonances at 2.5 ppm and 11.7 ppm are attributed to d_6_-DMSO and D_2_SO_4_, respectively.


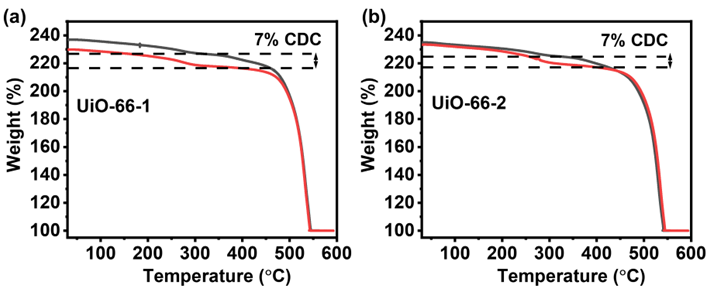


**Figure S7.** The TGA curves of UiO-66-1 (a) and UiO-66-2 (b) were shown before (black) and after (red) thermal decomposition of CDC.


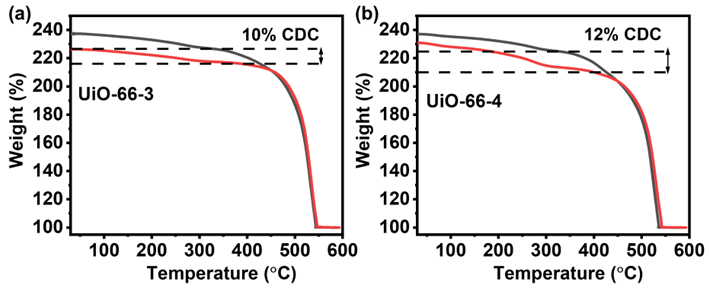


**Figure S8.** The TGA curves of UiO-66-3 (a) and UiO-66-4 (b) were shown before (black) and after (red) thermal decomposition of CDC.


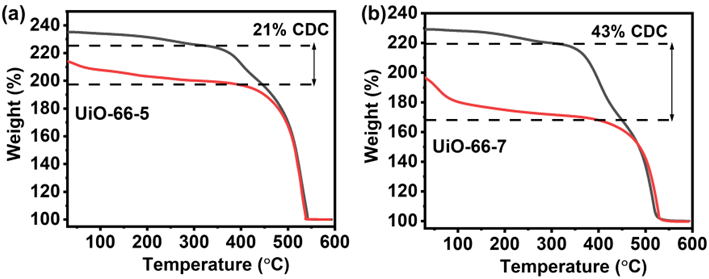


**Figure S9.** The TGA curves of UiO-66-5 (a) and UiO-66-7 (b) were shown before (black) and after (red) thermal decomposition of CDC.


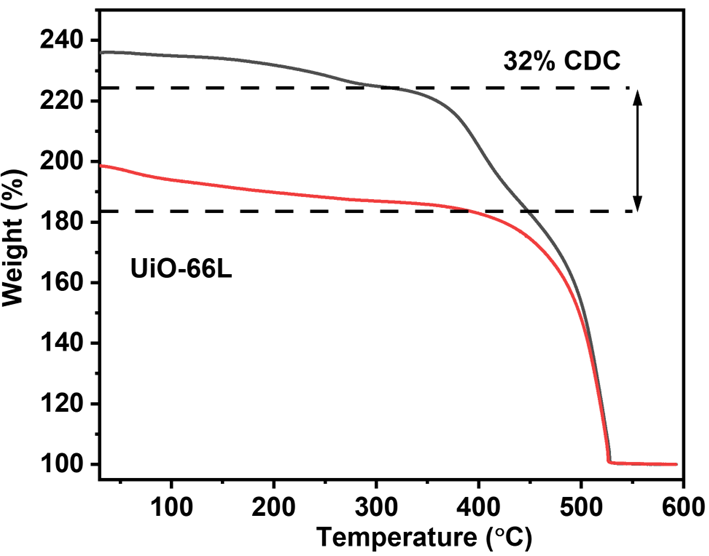


**Figure S10.** The TGA curves of UiO-66L was shown before (black) and after (red) thermal decomposition of CDC.

**Figure S11.** Unnormalized TGA data for UiO-66I, UiO-66L, and UiO-66C.


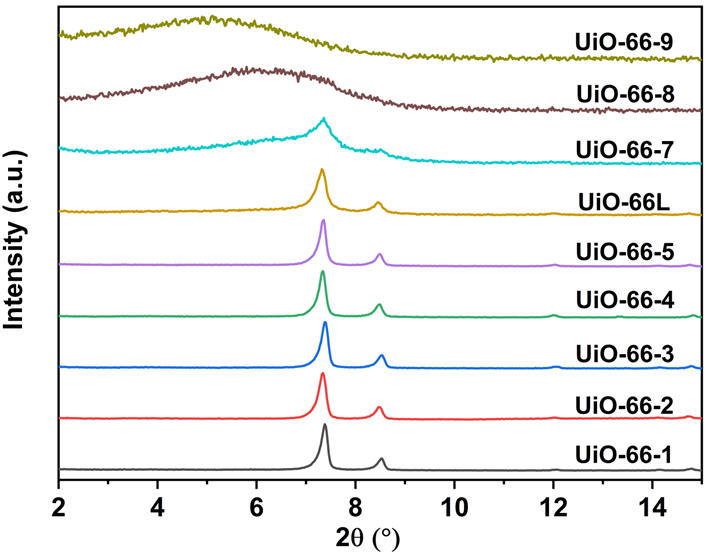


**Figure S12.** PXRD pattern of UiO-66 with different proportions of H_2_CDC after thermal decomposition of CDC by heating for 2 h in air at 325 °C.


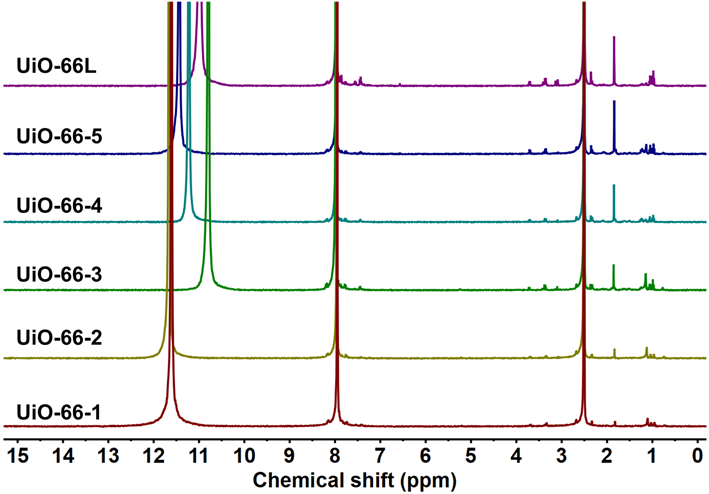


**Figure S13.** ^1^H NMR spectra of UiO-66 with different proportions of H_2_CDC after thermal decomposition. CDC signals disappeared at 1.2 ppm, 1.8 ppm, and 2.1 ppm in all samples.


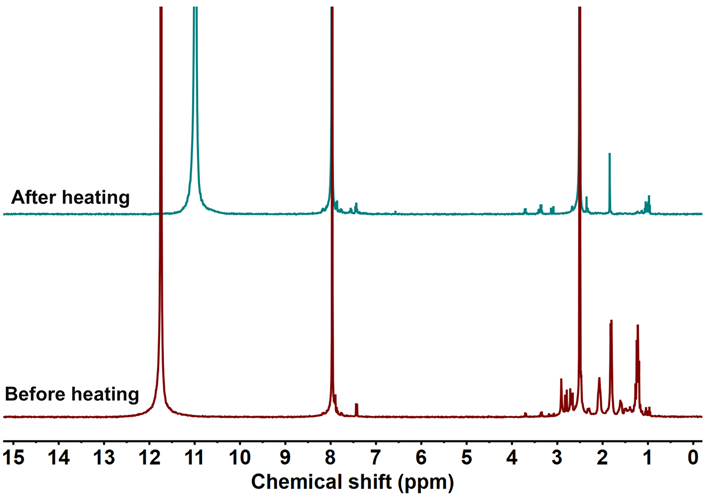


**Figure S14.** ^1^H NMR spectra of UiO-66L were shown before (red) and after (cyan) thermal decomposition of CDC.


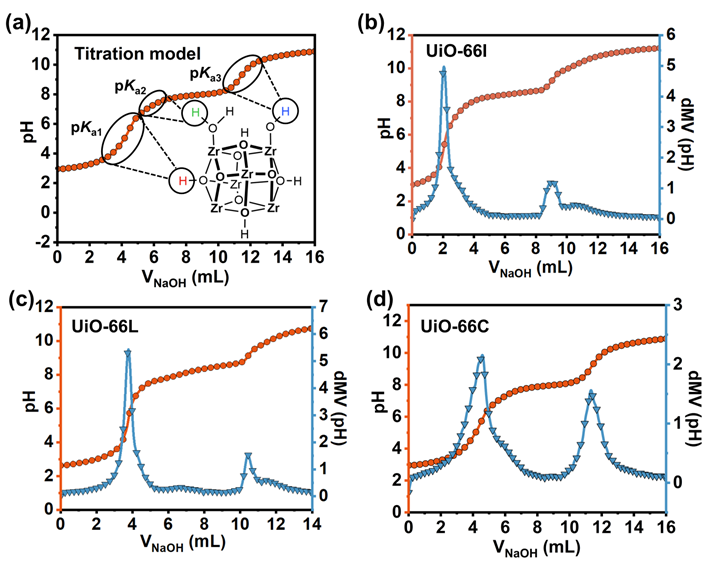


**Figure S15.** (a) Acid-base titration model of three protons (µ_3_-OH, Zr-OH_2_, and Zr-OH) on Zr_6_ cluster. Acid-base titration curve (red) and first derivative curve (blue) of (b) UiO-66I, (c) UiO-66L and (d) UiO-66C.


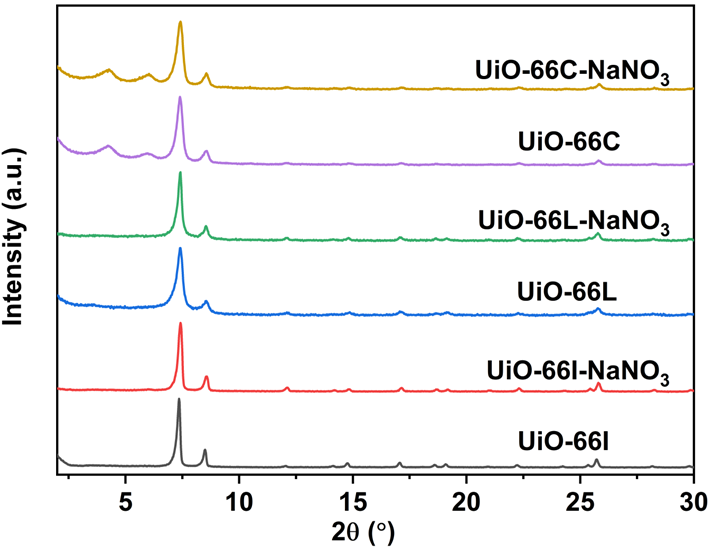


**Figure S16.** The PXRD pattern of UiO-66C, UiO-66L, and UiO-66I were shown after 18 h equilibrium in 0.01 M NaNO_3_ solution.


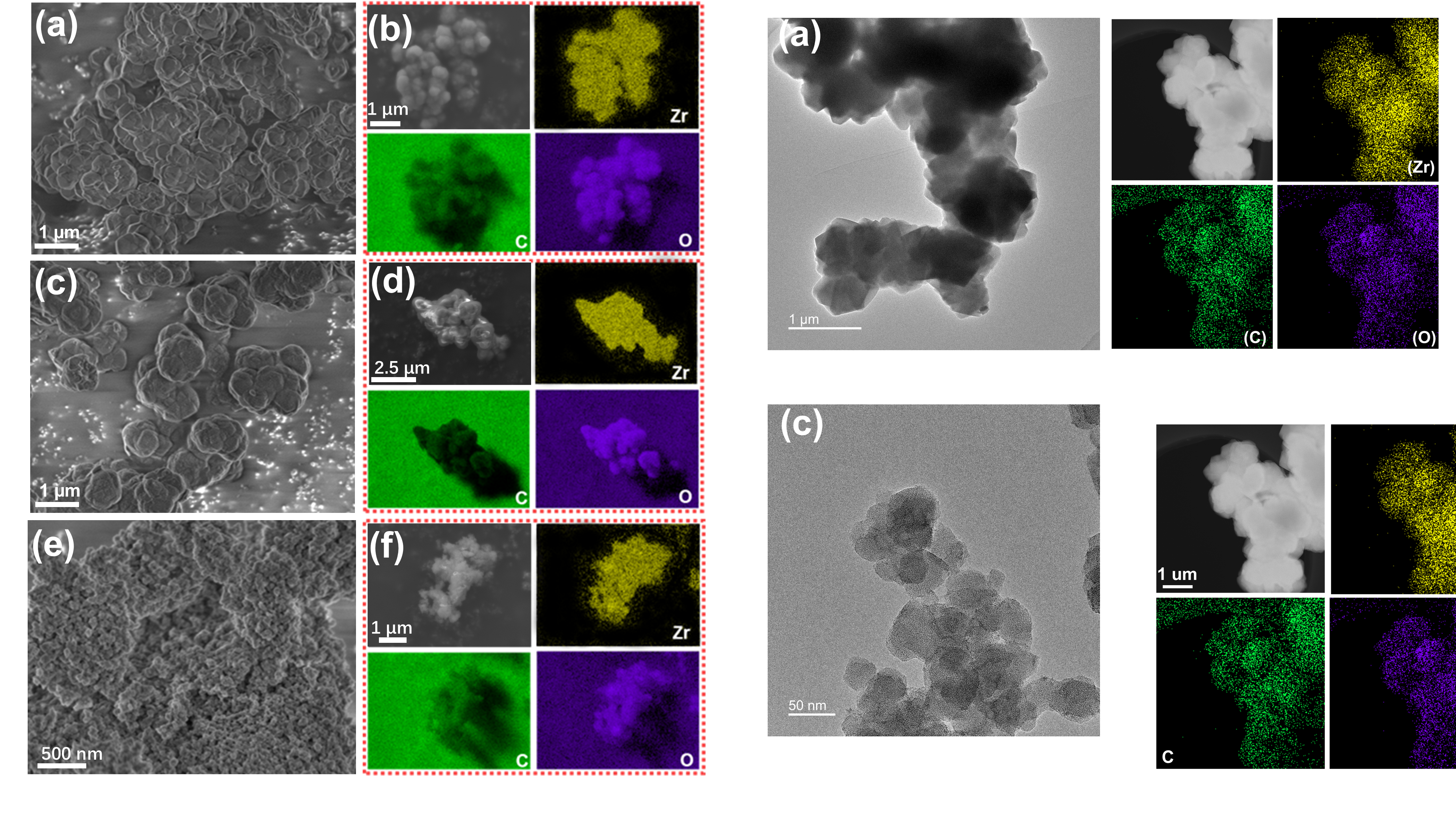


**Figure S17.** SEM images and element mapping of (a) and (b) UiO-66I, (c) and (d) UiO-66L, and (e) and (f) UiO-66C.


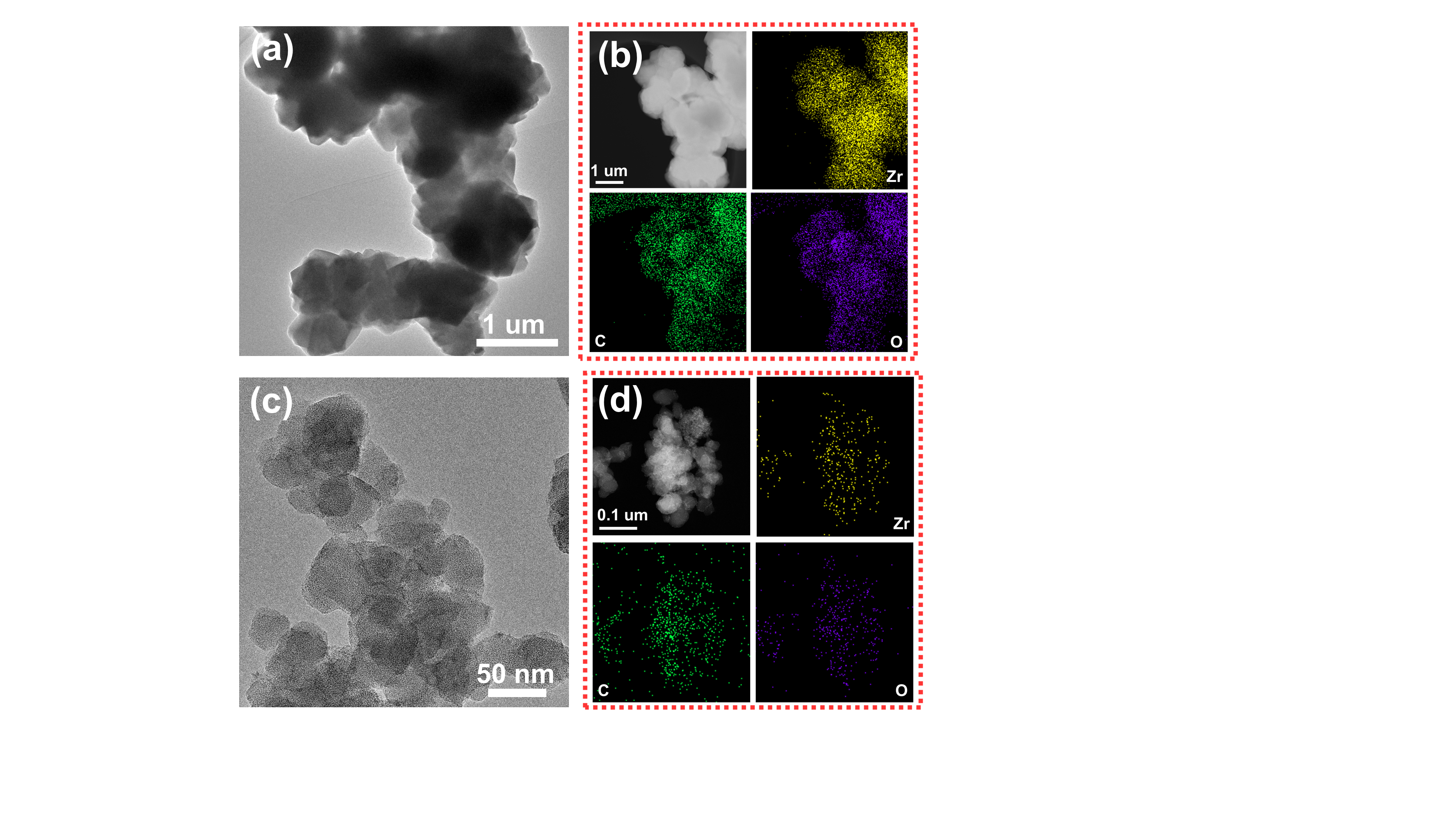


**Figure S18.** (a) TEM images and element mapping of (a) and (b) UiO-66L, (c) and (d) UiO-66C.


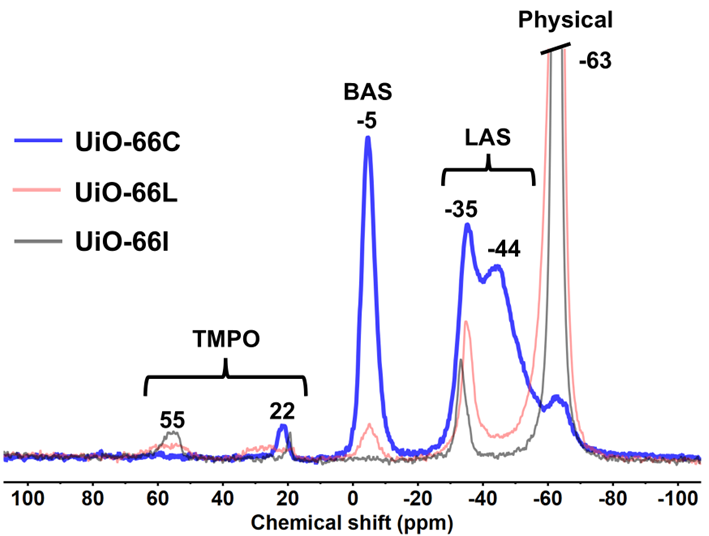


**Figure S19.** ^31^P MAS NMR spectra of TMP adsorbed on UiO-66I, UiO-66L and UiO-66C. ^31^P peaks observed at 55 and 22 ppm were attributed to trimethylphosphine oxide (TMPO) resulting from the partial oxidation of TMP molecules.


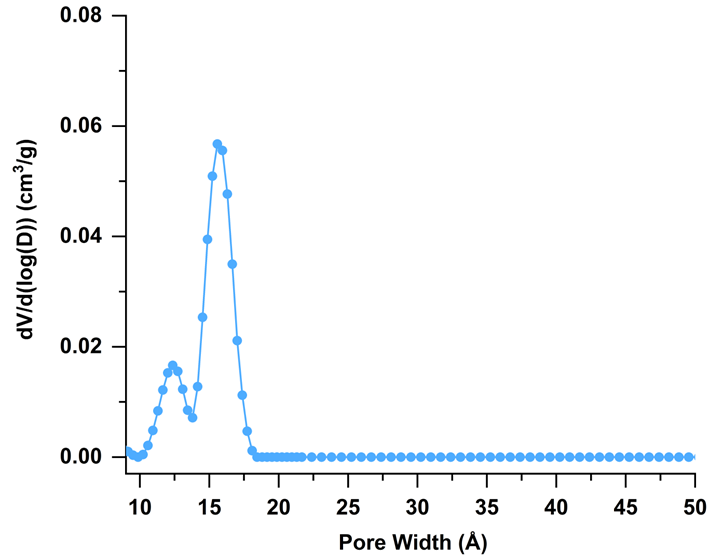


**Figure S20.** The pore size distribution of UIO-66C extended into the mesoporous range.


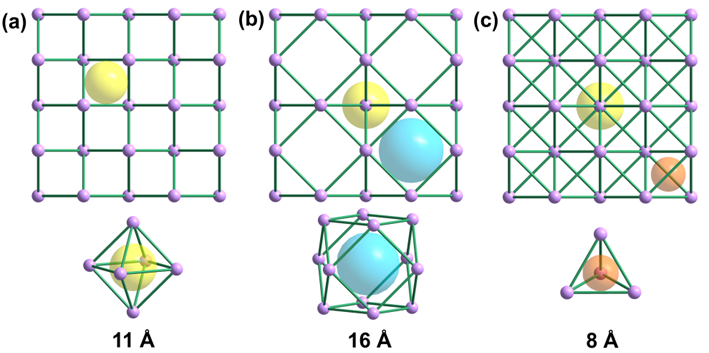


**Figure S21.** Pore size of (a) UiO-66L (*bcu* net), (b) UiO-66C (*reo* net), and (c) UiO-66I (*fcu* net).


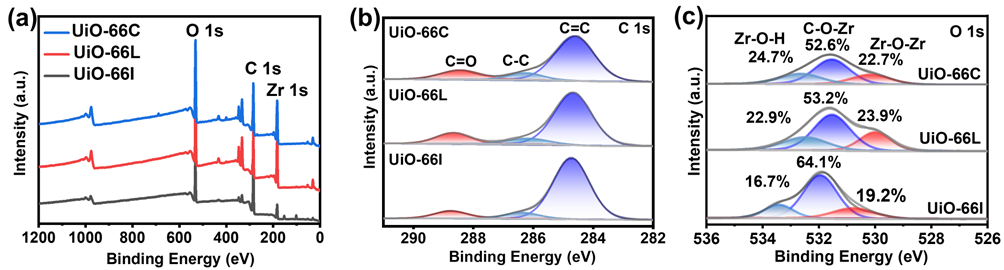


**Figure S22.** (a) XPS survey spectra and (b) C 1s spectra, (c) O1s spectra of UiO-66I, UiO-66L and UiO-66C.


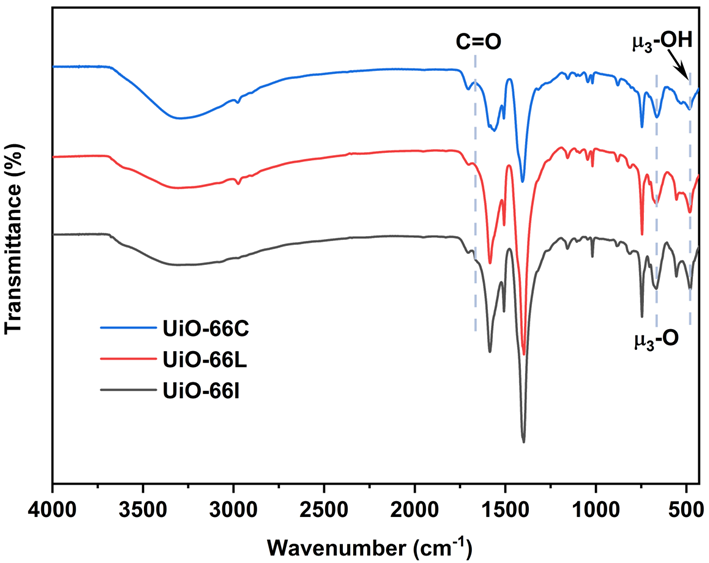


**Figure S23.** FTIR spectra of UiO-66C, UiO-66L and UiO-66I.


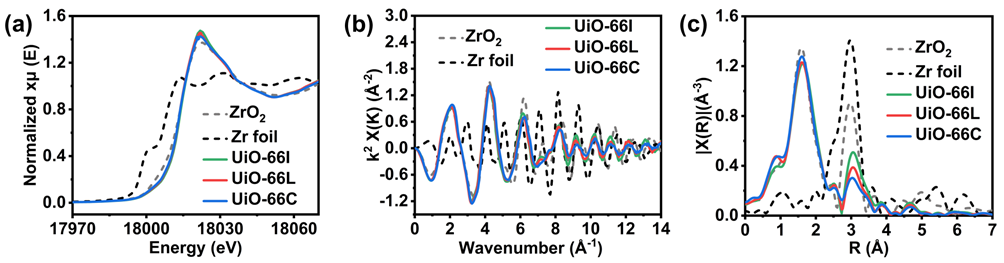


**Figure S24.** (a) Zr K-edge XANES spectra, (b) K space spectra, (c) Fourier transforms of k^3^-weighted EXAFS spectra of UiO-66I, UiO-66L and UiO-66C.


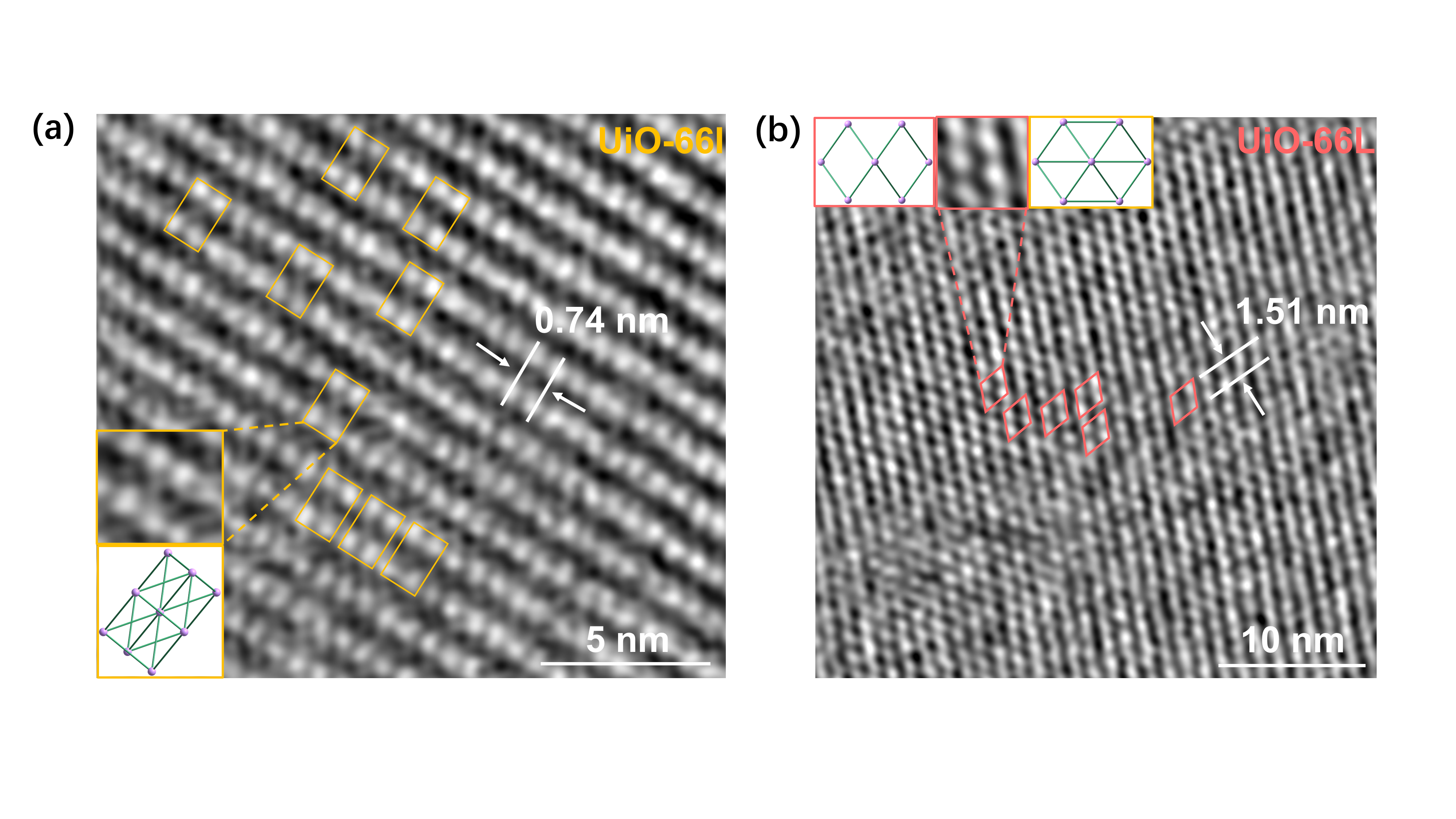


**Figure S25.** (a) iDPC-STEM image of UiO-66I and theoretical model of UiO-66I (inset) oriented along the 112 zone axis, (b) iDPC-STEM image of UiO-66L and theoretical model of UiO-66L oriented along the 101 zone axis (inset).


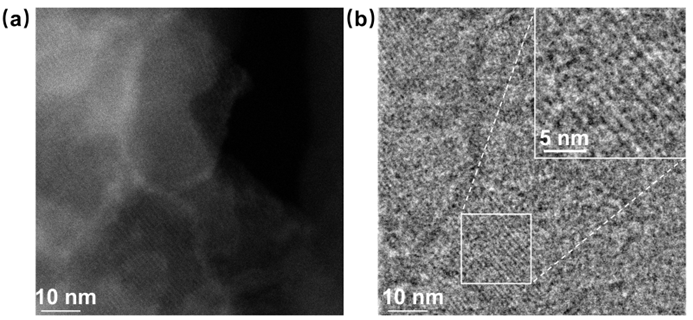


**Figure S26.** (a) HAADF-STEM image of UiO-66C, (b) iDPC-STEM image of UiO-66C and the lattice fringe (inset).


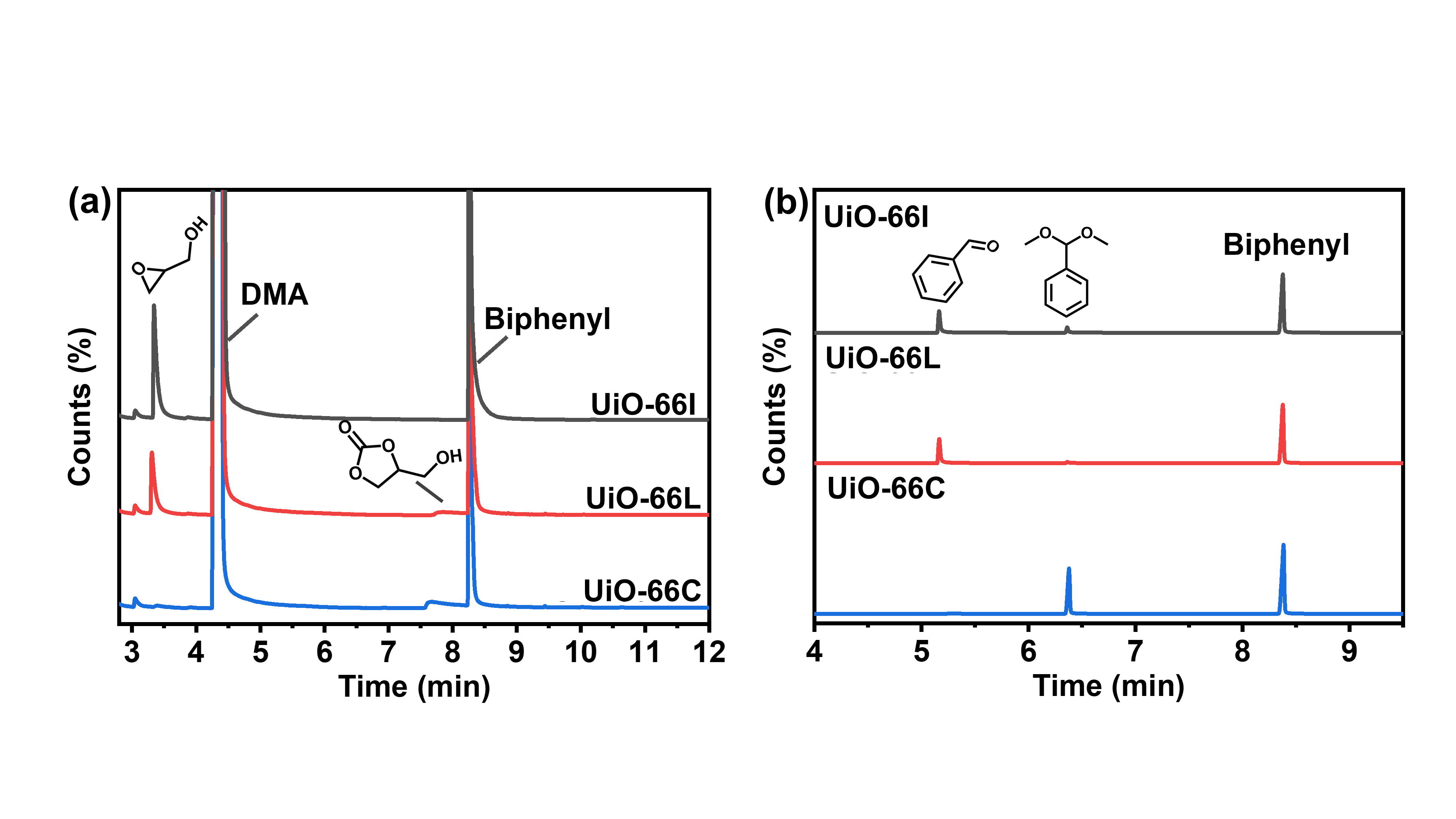


**Figure S27.** GC-MS chromatograms of (a) the cycloaddition reaction of CO_2_ and (b) acetalization of aldehydes.


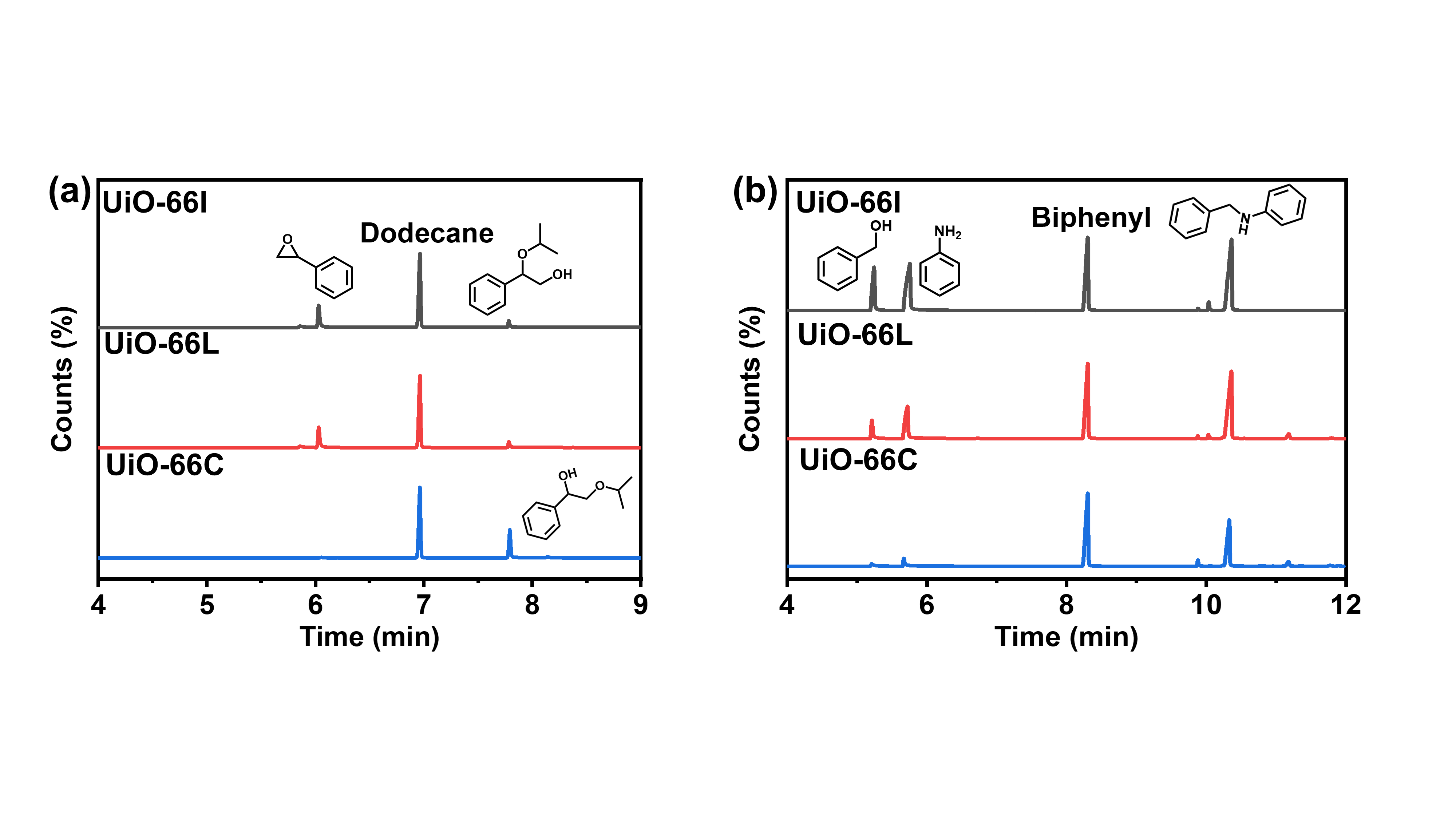


**Figure S28.** GC-MS chromatograms of (a) ring-opening reaction of styrene oxide and (b) N-alkylation.


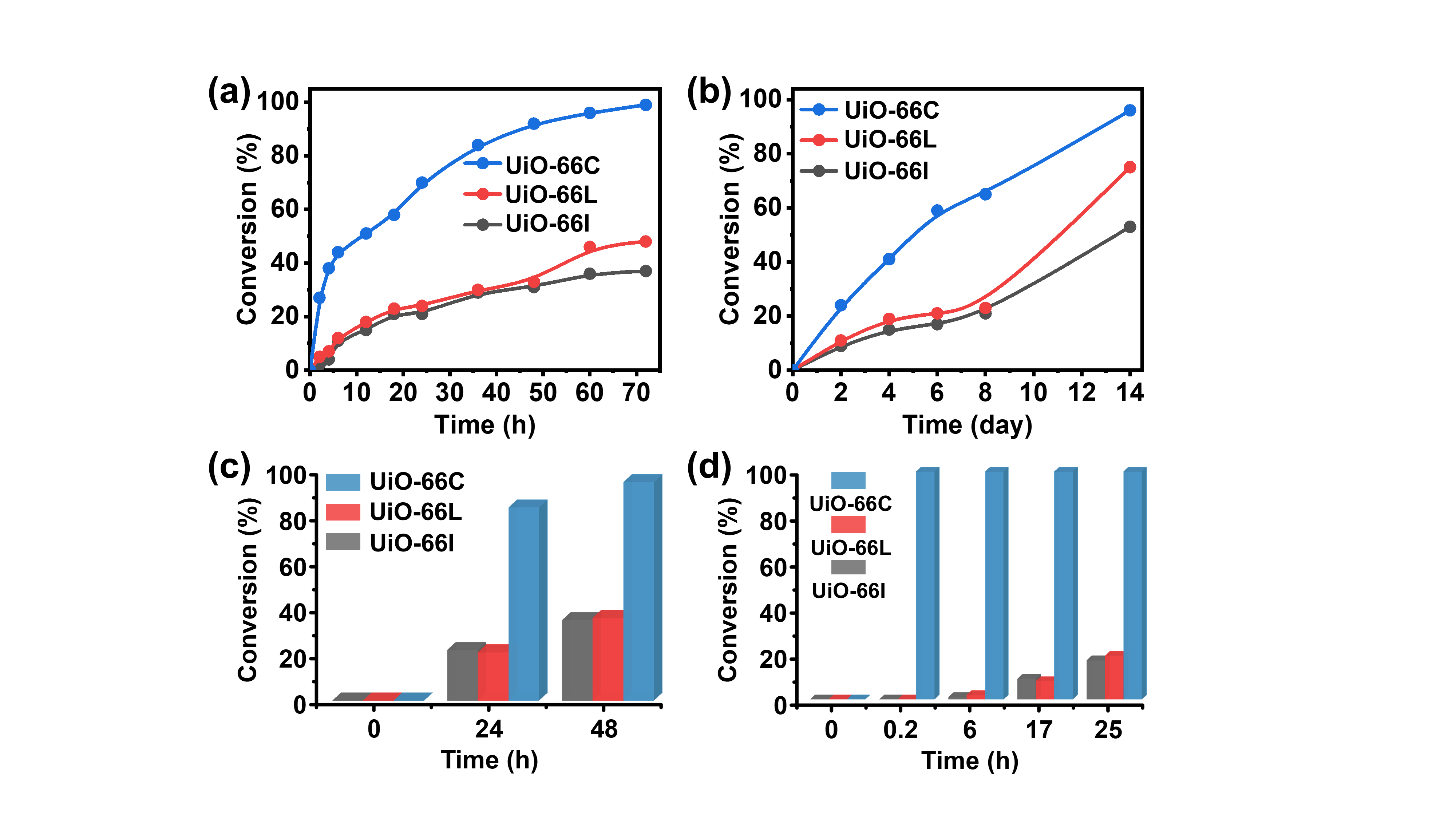


**Figure S29.** Conversion curves of (a) the cycloaddition reaction of CO_2_ and (b) N-alkylation, catalytic performance of (c) ring-opening reaction of styrene oxide and (d) acetalization of aldehydes.


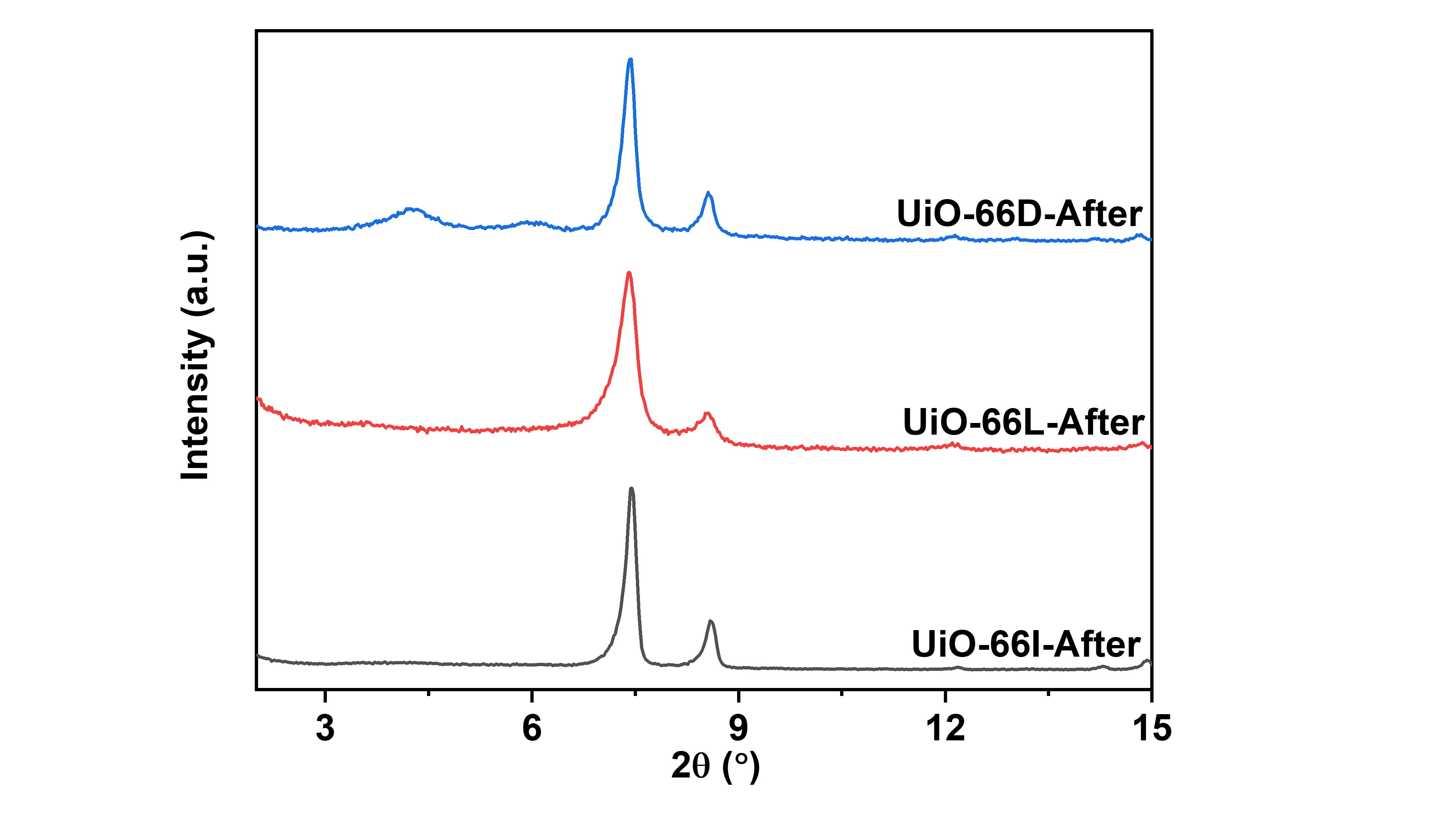


**Figure S30.** PXRD after the cycloaddition reaction of CO_2_.


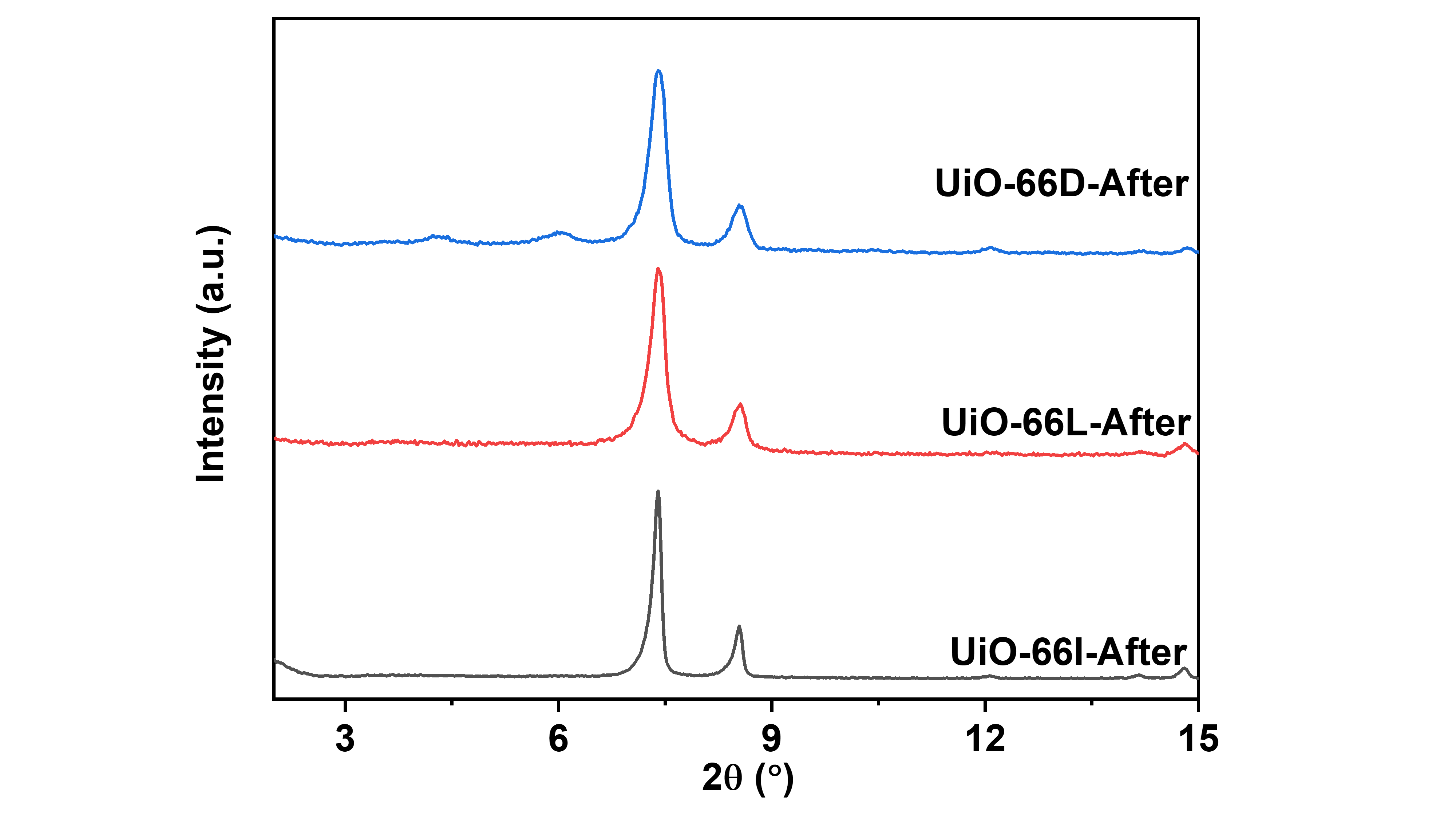


**Figure S31.** PXRD after the N-alkylation.


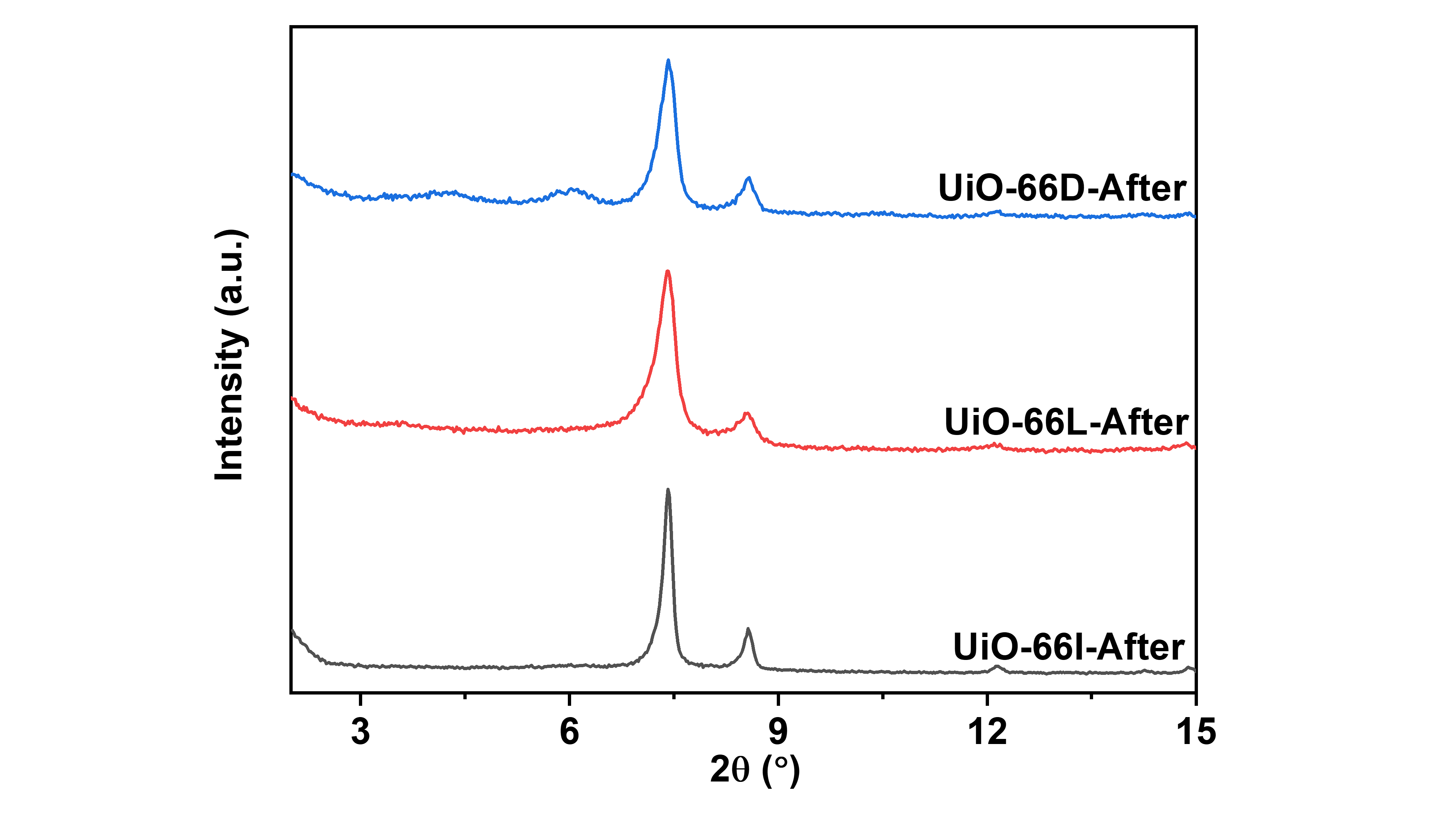


**Figure S32.** PXRD after the ring-opening reaction of styrene oxide.


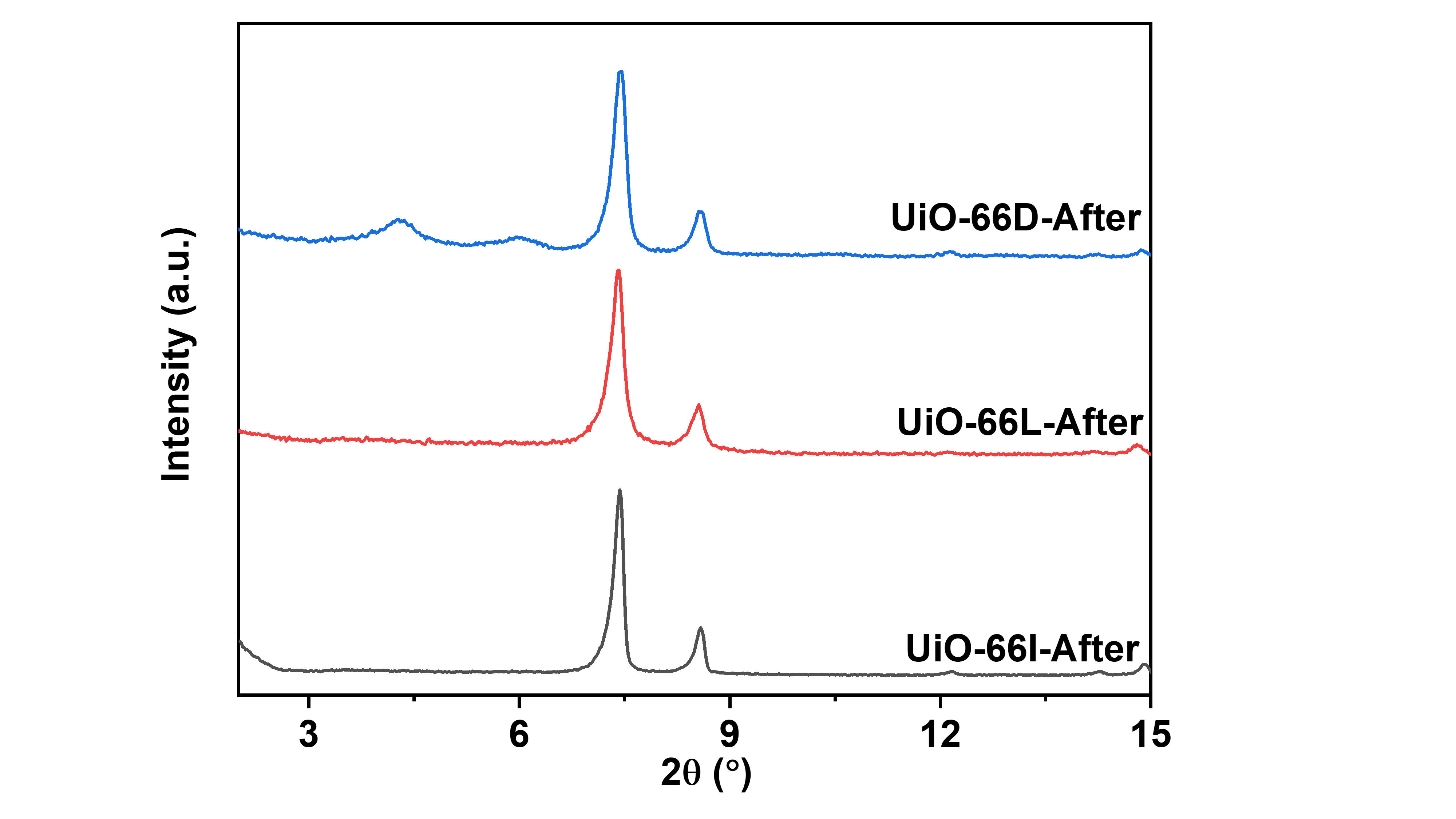


**Figure S33.** PXRD after the acetalization of aldehydes.


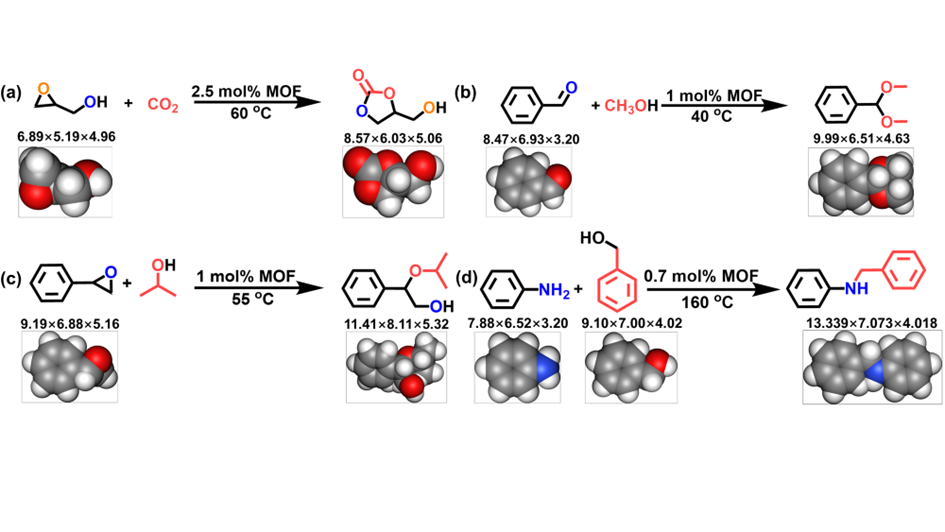


**Figure S34.** The molecular dimensions of substrates in the four catalytic models (a) the cycloaddition reaction of CO_2_, (b) acetalization of aldehydes, (c) ring-opening reaction of styrene oxide, (d) N-alkylation.

**
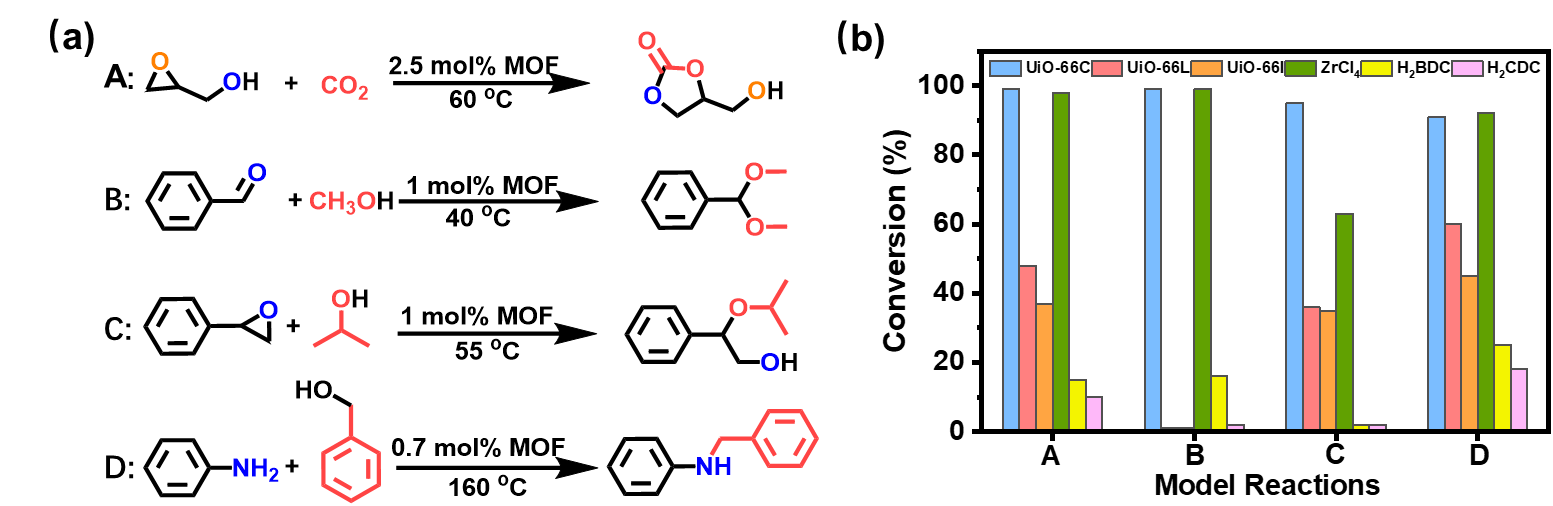
**

**Figure S35.** (a) Model catalytic reactions (A: the cycloaddition reaction of CO_2_, B: acetalization of aldehydes, C: ring-opening reaction of styrene oxide, D: N-alkylation), (b) conversion of four model reactions of UiO-66C, UiO-66L, UiO-66I, ZrCl_4_, H_2_BDC and H_2_CDC.

**Figure S36.** Catalytic mechanism of the cycloaddition reaction of CO_2_ on the MOFs catalysts (TS refers to the transition state).


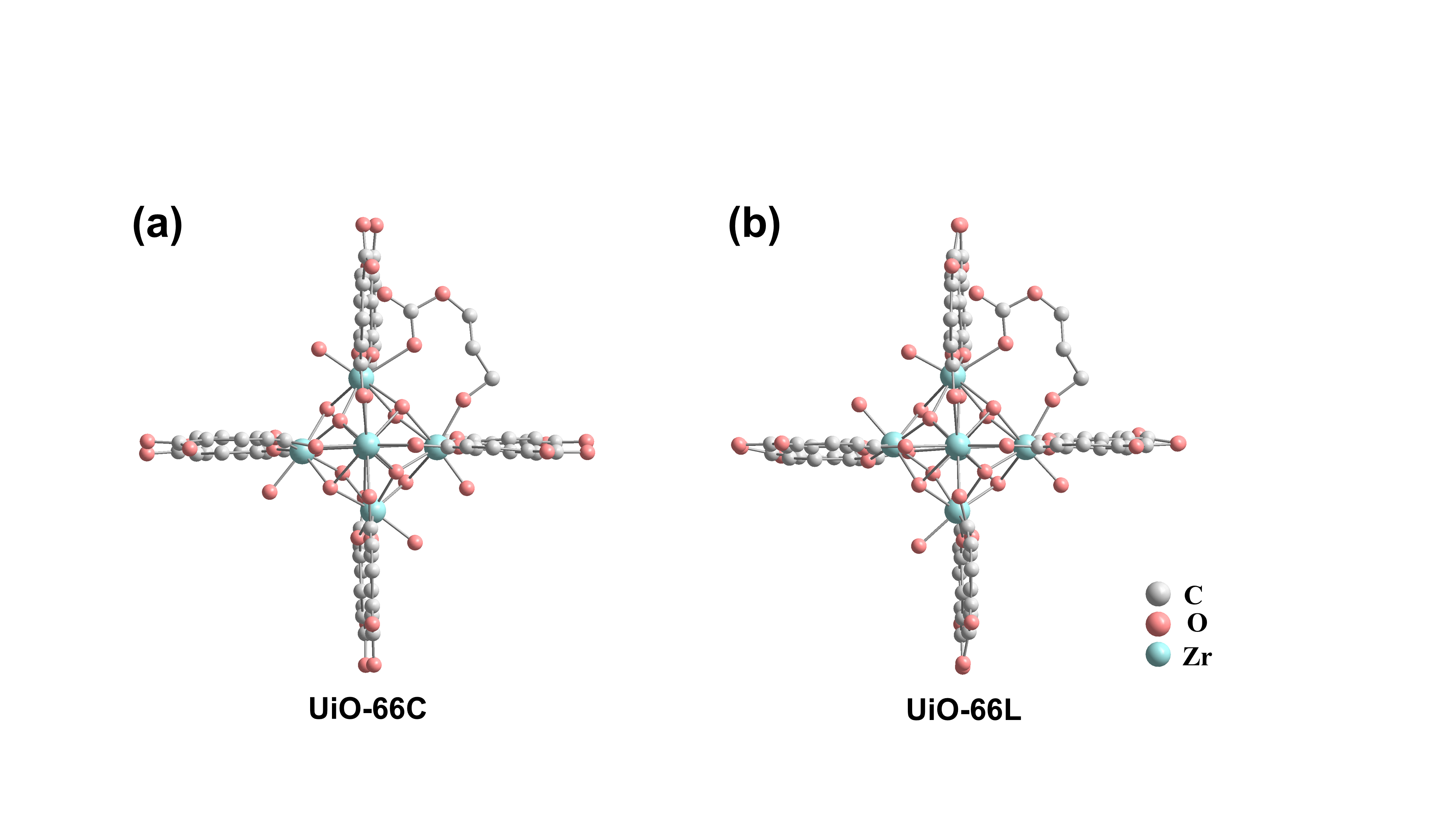


**Figure S37.** Transition state structures of epoxide ring-opening in the cycloaddition reaction of CO_2_ reaction catalyzed by (a) UiO-66C and (b) UiO-66L.


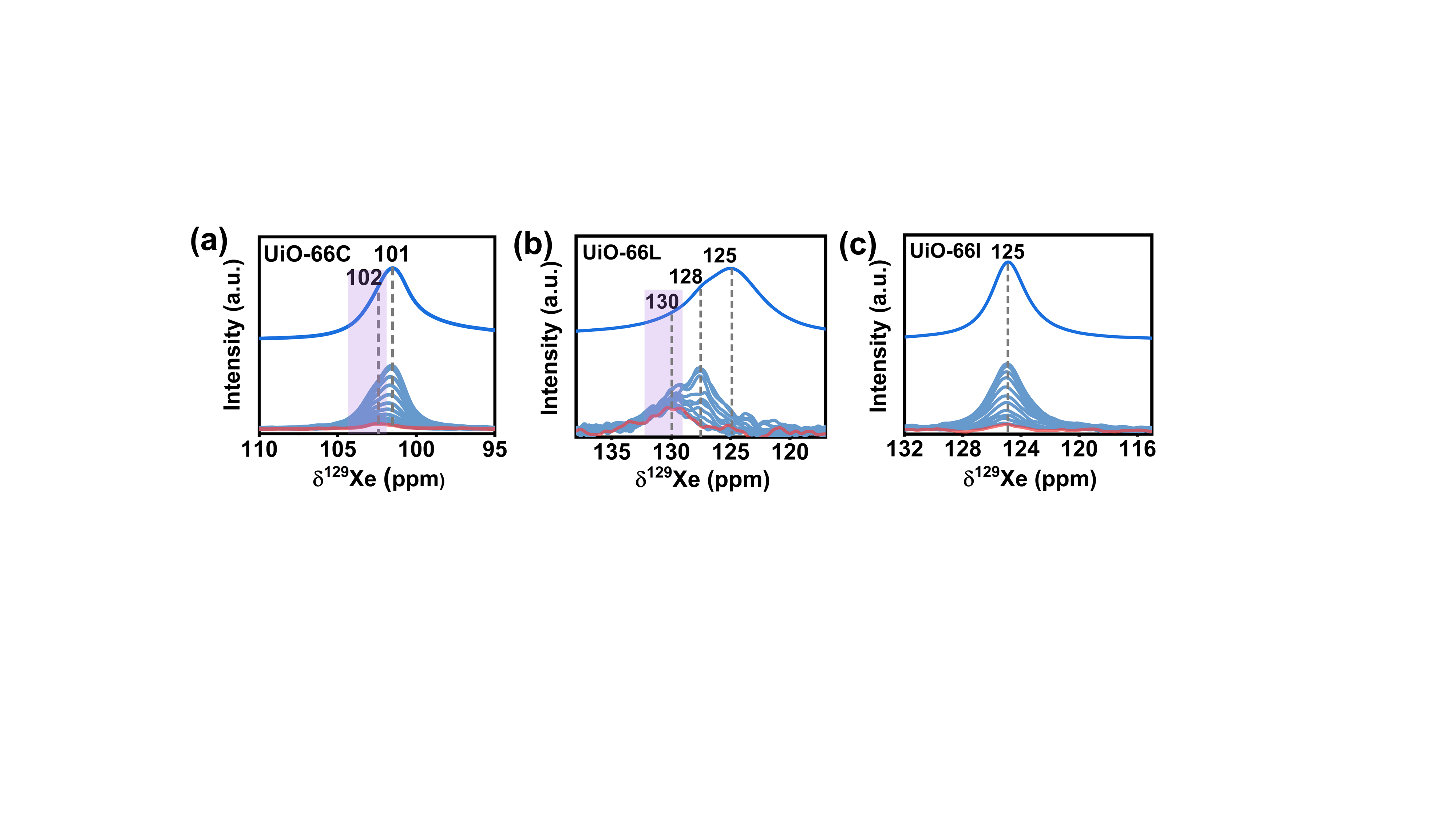


**Figure S38.** The top are the direct-excitation NMR spectra and the bottom are the PFG NMR spectra, and the red lines are the maximum gradient field intensity of (a) UiO-66C (g: 10→300 G/m), (b) UiO-66L (g: 10→300 G/m), and (c) UiO-66I (g: 10→140 G/m).


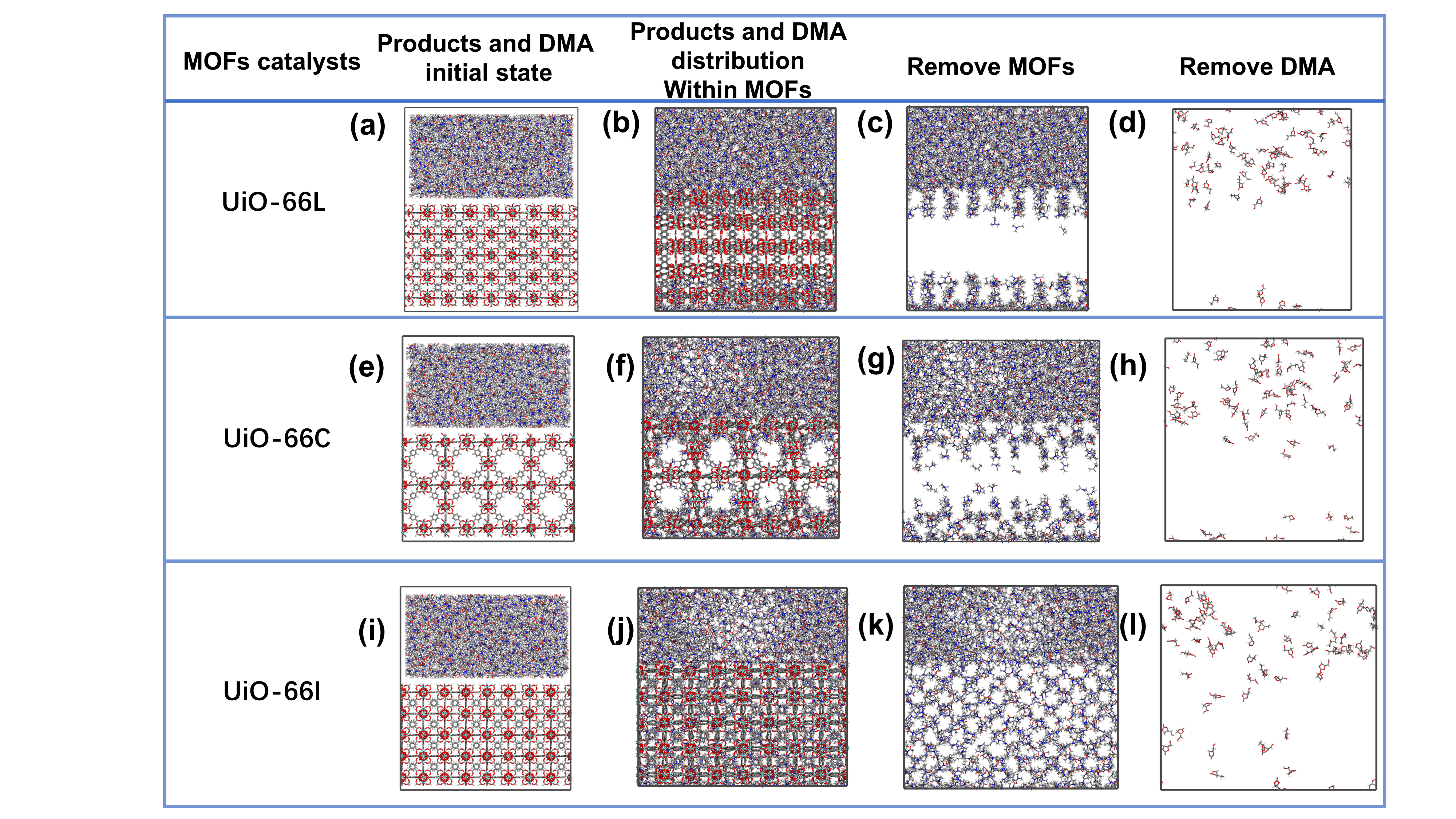


**Figure S39.** Molecular dynamics simulations of CO_2_ cycloaddition products diffusion, the initial states of the products and DMA before diffusion (a, e and i), products and the DMA distribution within MOFs (b, f and j), distribution of the products and DMA after the removal of the MOFs (c, g and k), distribution of the products after the removal of MOFs and DMA (d, h and l).


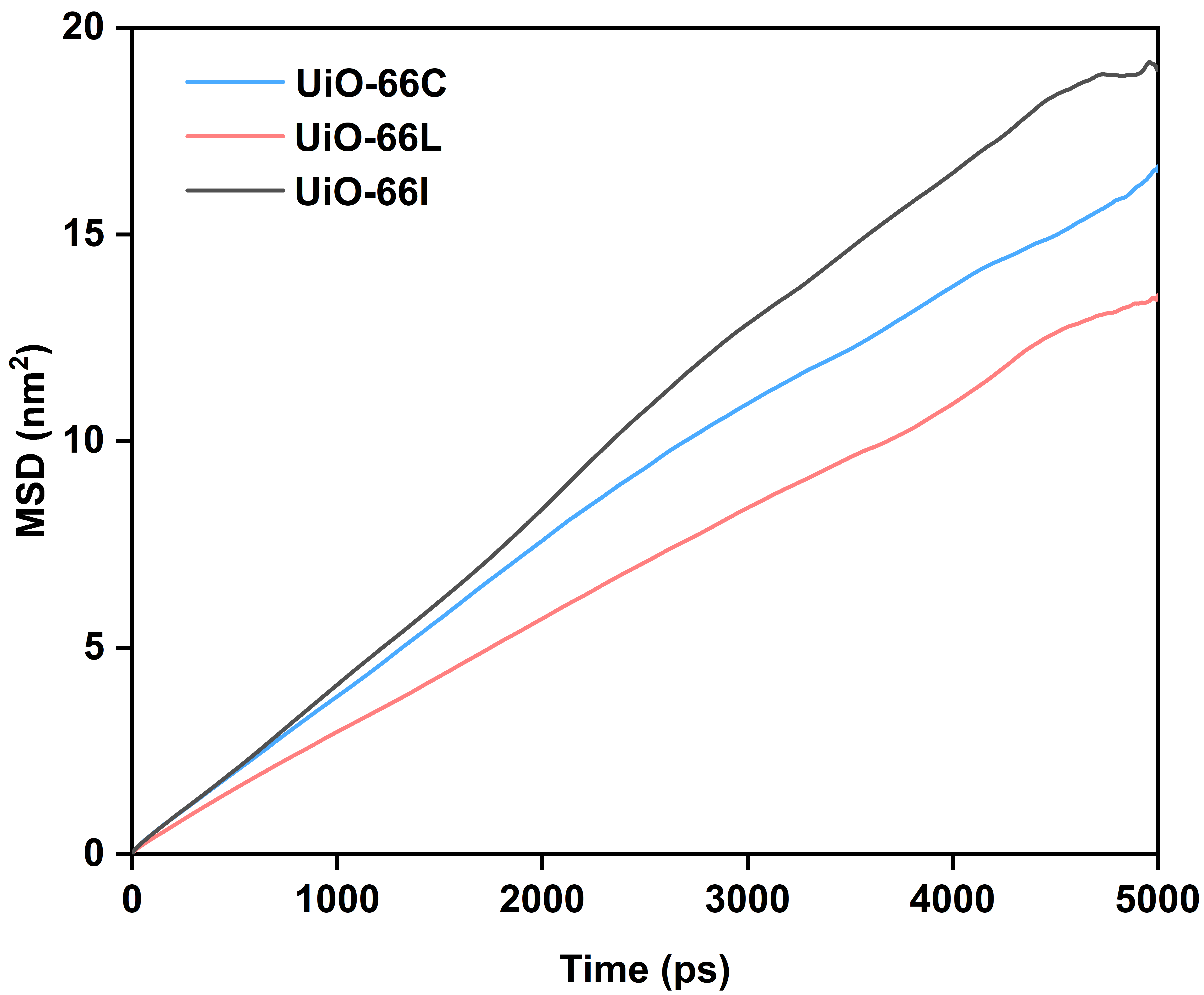


**Figure S40.** Mean-squared displacements of CO_2_ cycloaddition products entering the UiO-66C, UiO-66L, and UiO-66I frameworks.


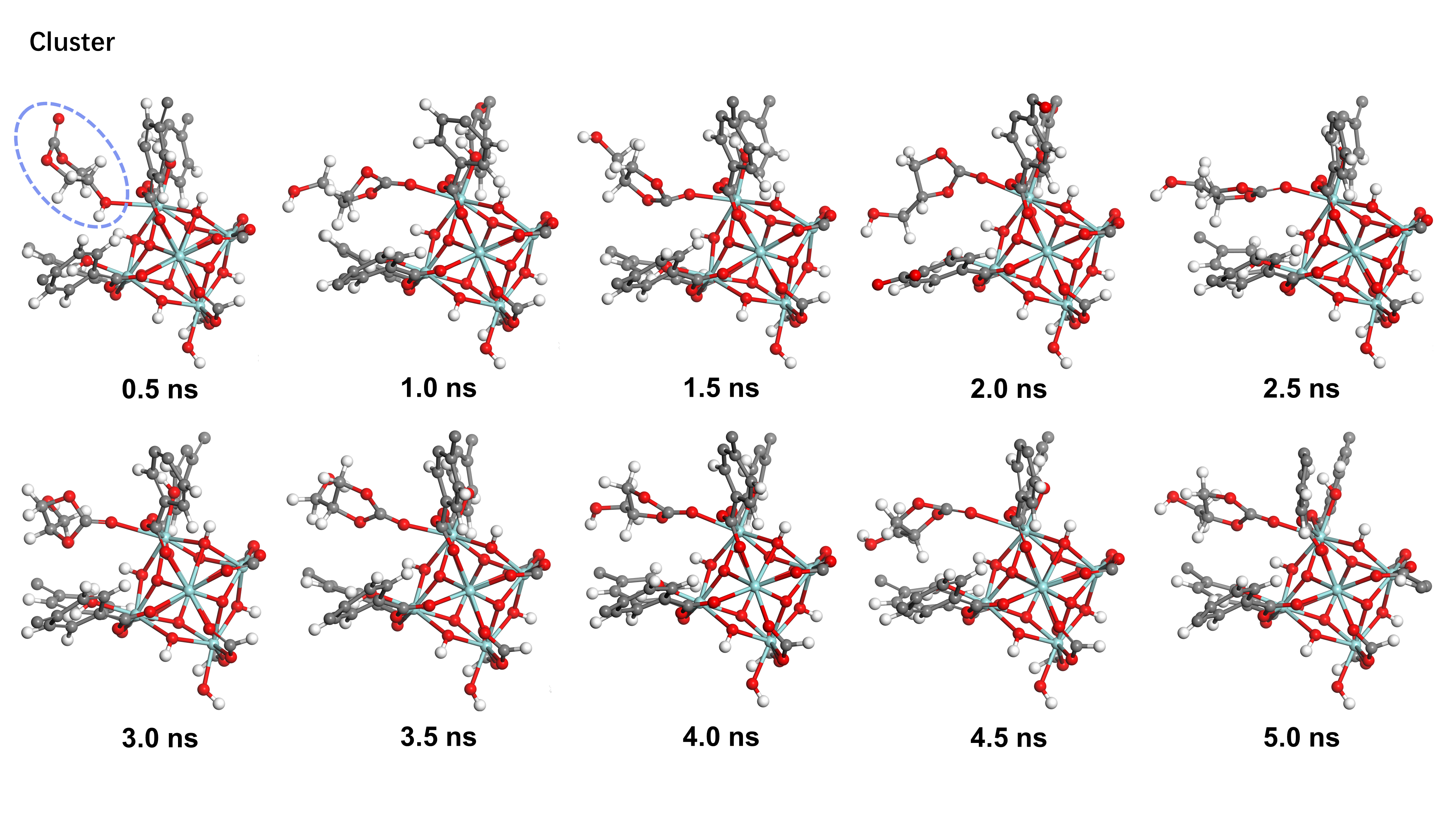


**Figure S41.** The molecular dynamics trajectories of adsorbed product molecule with open Zr sites in UiO-66C at different time.


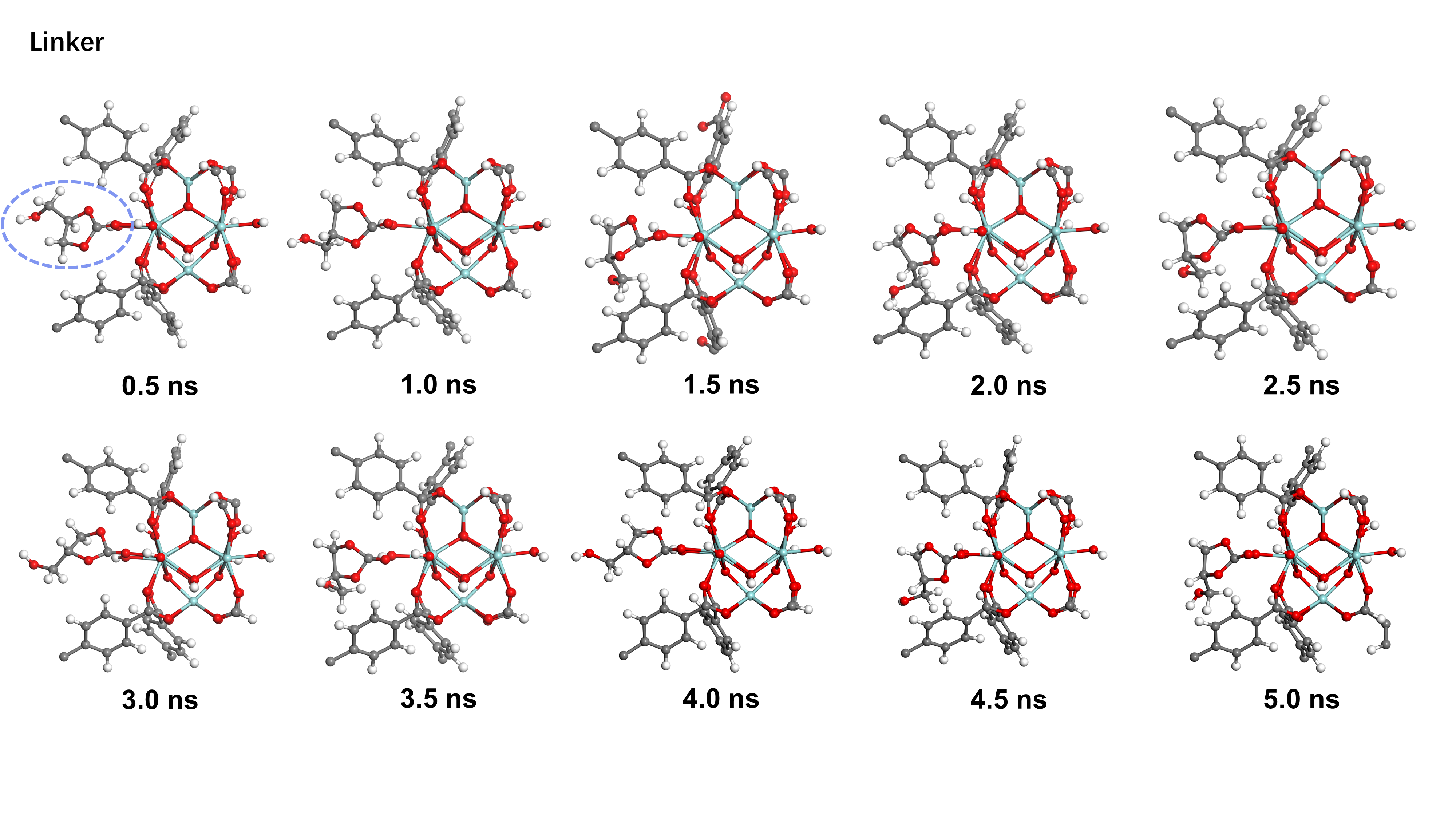


**Figure S42.** The molecular dynamics trajectories of adsorbed product molecule with open Zr sites in UiO-66L at different time.

Table S1. Metal ratios and absolute amounts employed in the synthesis of mixed-metal UiO-66, the number of defects after treatment with 0.1 M HCl.

| Sample | ZrCl_4_: Zn(NO_3_)_2_  (molar ratio) | ZrCl_4_  (mg) | ZrCl_4_ (mmol) | Zn(NO_3_)_2_ (mg) | Zn(NO_3_)_2_ (mmol) | Defects |
| --- | --- | --- | --- | --- | --- | --- |
| UiO-66-10 | 1:0.33 | 470 | 2.02 | 200 | 0.67 | 2.2 |
| UiO-66-11 | 1:0.5 | 470 | 2.02 | 300 | 1.01 | 2.6 |
| UiO-66-12 | 1:0.67 | 470 | 2.02 | 402 | 1.35 | 2.8 |
| UiO-66C | 1:1 | 470 | 2.02 | 600 | 2.02 | 3.6 |

Table S2. Linker ratios and absolute amounts employed in the synthesis of mixed-linker UiO-66.

| Sample | H_2_BDC:H_2_CDC  (molar ratio) | H_2_BDC (mg) | H_2_BDC (mmol) | H_2_CDC (mg) | H_2_CDC (mmol) |
| --- | --- | --- | --- | --- | --- |
| UiO-66-1 | 9:1 | 603 | 3.63 | 69 | 0.40 |
| UiO-66-2 | 8:2 | 536 | 3.23 | 139 | 0.81 |
| UiO-66-3 | 7:3 | 469 | 2.82 | 208 | 1.21 |
| UiO-66-4 | 6:4 | 402 | 2.42 | 278 | 1.61 |
| UiO-66-5 | 5:5 | 335 | 2.02 | 347 | 2.02 |
| UiO-66L | 4:6 | 268 | 1.61 | 417 | 2.42 |
| UiO-66-7 | 3:7 | 201 | 1.21 | 486 | 2.82 |
| UiO-66-8 | 2:8 | 134 | 0.81 | 556 | 3.23 |
| UiO-66-9 | 1:9 | 67 | 0.40 | 625 | 3.63 |

Table S3. The mole fraction of CDC relative to the total amount of linkers incorporated in the obtained frameworks, as determined by ^1^H NMR and TGA. PXRD of UiO-66 with different CDC content before and after heating and the number of defects after thermal decomposition CDC.

| Sample | H_2_CDC in NMR (%) | H_2_CDC in TGA (%) | PXRD before heating | PXRD after heating | Defects |
| --- | --- | --- | --- | --- | --- |
| UiO-66-1 | 5 | 7 | √ | √ | 0.4 |
| UiO-66-2 | 6 | 7 | √ | √ | 0.4 |
| UiO-66-3 | 9 | 10 | √ | √ | 0.6 |
| UiO-66-4 | 12 | 12 | √ | √ | 1.0 |
| UiO-66-5 | 19 | 21 | √ | √ | 2.4 |
| UiO-66L | 29 | 32 | √ | √ | 3.6 |
| UiO-66-7 | 42 | 43 | √ | √ | 5.2 |
| UiO-66-8 | 59 | - | √ | × | - |
| UiO-66-9 | 74 | - | √ | × | - |

Table S4. NaOH volume (V_NaOH_) and corresponding acidity between different jumps in UiO-66I, UiO-66L and UiO-66C.

| Samples | Range of p*K*_a_ | V_NaOH_ (mL) | Acidity (mmol/g) |
| --- | --- | --- | --- |
| UiO-66C | ～p*K*_a1_ | 4.6 | 3.22 |
|  | p*K*_a1_～p*K*_a2_ | 1.3 | 1.00 |
|  | p*K*_a2_～p*K*_a3_ | 5.4 | 3.80 |
|  | p*K*_a1_～p*K*_a3_ | 6.7 | 4.60 |
|  | total | 11.3 | 7.92 |
| UiO-66L | ～p*K*_a1_ | 3.80 | 2.66 |
|  | p*K*_a1_～p*K*_a2_ | 2.90 | 2.00 |
|  | p*K*_a2_～p*K*_a3_ | 3.70 | 2.60 |
|  | p*K*_a1_～p*K*_a3_ | 6.60 | 4.60 |
|  | total | 10.40 | 7.28 |
| UiO-66I | ～p*K*_a1_ | 2.00 | 1.40 |
|  | p*K*_a1_～p*K*_a2_ | - | - |
|  | p*K*_a2_～p*K*_a3_ | - | - |
|  | p*K*_a1_～p*K*_a3_ | - | - |
|  | total | - | - |

Table S5. BET surface area, pore size (nm) and pore volume of samples.

| Samples | BET surface area (m^2^/g) | Pore size (nm) | Pore volume (cm^3^/g) |
| --- | --- | --- | --- |
| UiO-66I | 1050 | 1.1 | 0.43 |
| UiO-66L | 907 | 1.1 | 0.39 |
| UiO-66C | 1227 | 1.2, 1.6 | 1.02 |

Table S6. The conversion of the four model reactions.

| Samples | The cycloaddition reaction of CO_2_ | Acetalization of aldehydes | Ring-opening reaction of styrene oxide (%) | N-alkylation (%) |
| --- | --- | --- | --- | --- |
| UiO-66I | 37 (72 h) | <1 (0.2 h) | 35 (48 h) | 45 (14 day) |
| UiO-66L | 48 (72 h) | <1 (0.2 h) | 36 (48 h) | 60 (14 day) |
| UiO-66C | 99 (72 h) | 99 (0.2 h) | 95 (48 h) | 91 (14 day) |
| ZrCl_4_ | 98 (72 h) | 99 (0.2 h) | 63 (48 h) | 92 (10 day) |
| H_2_BDC | 15 (72 h) | 16 (0.2 h) | <5 (48 h) | 25 (10 day) |
| H_2_CDC | 10 (72 h) | <5 (0.2 h) | <5 (48 h) | 18 (10 day) |
| Blank | <5 (72 h) | <5 (0.2 h) | <5 (48 h) | 10 (14 day) |

Table S7. Turnover Number (TON) of different UiO-66 samples in four catalytic models (calculated based on the Zr_6_ cluster).

| Samples | The cycloaddition reaction of CO_2_ | Acetalization of aldehydes | Ring-opening reaction of styrene oxide | N-alkylation |
| --- | --- | --- | --- | --- |
| UiO-66I | 14.23 | 17.24 | 33.33 | 82.81 |
| UiO-66L | 16.00 | 15.76 | 28.24 | 96.15 |
| UiO-66C | 33.00 | 82.13 | 74.51 | 123.08 |

Table S8. Turnover Frequency (TOF) (h^-1^) of different UiO-66 samples in three catalytic models (calculated based on the Zr_6_ cluster).

| Samples | The cycloaddition reaction of CO_2_ | Acetalization of aldehydes | Ring-opening reaction of styrene oxide | N-alkylation |
| --- | --- | --- | --- | --- |
| UiO-66I | 0.38 | 0.48 | 1.90 | 0.29 |
| UiO-66L | 0.83 | 0.49 | 2.16 | 0.29 |
| UiO-66C | 4.50 | 4.83 | 4.90 | 0.64 |

**References**

[1] Ravel, B., Newville, M., *ATHENA*, *ARTEMIS*, *HEPHAESTUS*: data analysis for X-ray absorption spectroscopy using *IFEFFIT*, 2005, J. Synchrotron Rad., 12, 537-541, doi:10.1107/S0909049505012719

[2] Kärger, J., Valiullin, R., Mass transfer in mesoporous materials: the benefit of microscopic diffusion measurement, 2013, Chem. Soc. Rev., 42, 4172-4197, 10.1039/C3CS35326E

[3] Cotts, R. M., Hoch, M. J. R., Sun, T., Markert, J. T., Pulsed field gradient stimulated echo methods for improved NMR diffusion measurements in heterogeneous systems, 1989, J. Magn. Reson., 83, 252-266, 10.1016/0022-2364(89)90189-3

[4] Pagès, G., Gilard, V., Martino, R., Malet-Martino, M., Pulsed-field gradient nuclear magnetic resonance measurements (PFG NMR) for diffusion ordered spectroscopy (DOSY) mapping, 2017, Analyst, 142, 3771-3796, 10.1039/C7AN01031A

[5] Achar, S. K., Wardzala, J. J., Bernasconi, L., Zhang, L., Johnson, J. K., Combined Deep Learning and Classical Potential Approach for Modeling Diffusion in UiO-66, 2022, J. Chem. Theory Comput., 18, 3593-3606, 10.1021/acs.jctc.2c00010

[6] Kresse, G., Hafner, J., Ab initio molecular-dynamics simulation of the liquid-metal-amorphous-semiconductor transition in germanium, 1994, Phys. Rev. B, 49, 14251-14269, 10.1103/PhysRevB.49.14251

[7] Perdew, J. P., Chevary, J. A., Vosko, S. H., Jackson, K. A., Pederson, M. R., Singh, D. J., Fiolhais, C., Atoms, molecules, solids, and surfaces: Applications of the generalized gradient approximation for exchange and correlation, 1992, Phys. Rev. B, 46, 6671-6687, 10.1103/PhysRevB.46.6671

[8] Kresse, G., Furthmüller, J., Efficiency of ab-initio total energy calculations for metals and semiconductors using a plane-wave basis set, 1996, Comput. Mater. Sci., 6, 15-50, 10.1016/0927-0256(96)00008-0

[9] Blöchl, P. E., Projector augmented-wave method, 1994, Phys. Rev. B, 50, 17953-17979, 10.1103/PhysRevB.50.17953

[10] Perdew, J. P., Burke, K., Ernzerhof, M., Generalized Gradient Approximation Made Simple, 1996, Phys. Rev. Lett., 77, 3865-3868, 10.1103/PhysRevLett.77.3865

[11] Grimme, S., Antony, J., Ehrlich, S., Krieg, H., A consistent and accurate ab initio parametrization of density functional dispersion correction (DFT-D) for the 94 elements H-Pu, 2010, J. Chem. Phys., 132, 154104, 10.1063/1.3382344

[12] Mathew, K., Sundararaman, R., Letchworth-Weaver, K., Arias, T. A., Hennig, R. G., Implicit solvation model for density-functional study of nanocrystal surfaces and reaction pathways, 2014, J. Chem. Phys., 140, 084106, 10.1063/1.4865107

[13] Mathew, K., Kolluru, V. S. C., Mula, S., Steinmann, S. N., Hennig, R. G., Implicit self-consistent electrolyte model in plane-wave density-functional theory, 2019, J. Chem. Phys., 151, 234101, 10.1063/1.5132354

[14] Yang, Q., Guillerm, V., Ragon, F., Wiersum, A. D., Llewellyn, P. L., Zhong, C., Devic, T., Serre, C., Maurin, G., CH_4_ storage and CO_2_ capture in highly porous zirconium oxide based metal–organic frameworks, 2012, Chem. Commun., 48, 9831-9833, 10.1039/C2CC34714H

[15] Cavka, J. H., Jakobsen, S., Olsbye, U., Guillou, N., Lamberti, C., Bordiga, S., Lillerud, K. P., A New Zirconium Inorganic Building Brick Forming Metal Organic Frameworks with Exceptional Stability, 2008, J. Am. Chem. Soc., 130, 13850-13851, 10.1021/ja8057953

[16] Valenzano, L., Civalleri, B., Chavan, S., Bordiga, S., Nilsen, M. H., Jakobsen, S., Lillerud, K. P., Lamberti, C., Disclosing the Complex Structure of UiO-66 Metal Organic Framework: A Synergic Combination of Experiment and Theory, 2011, Chem. Mat., 23, 1700-1718, 10.1021/cm1022882

[17] Wang, H.-F., Liu, Z.-P., Comprehensive Mechanism and Structure-Sensitivity of Ethanol Oxidation on Platinum: New Transition-State Searching Method for Resolving the Complex Reaction Network, 2008, J. Am. Chem. Soc., 130, 10996-11004, 10.1021/ja801648h

[18] Henkelman, G., Uberuaga, B. P., Jónsson, H., A climbing image nudged elastic band method for finding saddle points and minimum energy paths, 2000, J. Chem. Phys., 113, 9901-9904, 10.1063/1.1329672

[19] Abraham, M. J., Murtola, T., Schulz, R., Páll, S., Smith, J. C., Hess, B., Lindahl, E., GROMACS: High performance molecular simulations through multi-level parallelism from laptops to supercomputers, 2015, SoftwareX, 1, 19-25, 10.1016/j.softx.2015.06.001

[20] Rappe, A. K., Casewit, C. J., Colwell, K. S., Goddard, W. A., III, Skiff, W. M., UFF, a full periodic table force field for molecular mechanics and molecular dynamics simulations, 1992, J. Am. Chem. Soc., 114, 10024-10035, 10.1021/ja00051a040

[21] Bussi, G., Donadio, D., Parrinello, M., Canonical sampling through velocity rescaling, 2007, J. Chem. Phys., 126, 014101, 10.1063/1.2408420

[22] Berendsen, H. J. C., Postma, J. P. M., van Gunsteren, W. F., DiNola, A., Haak, J. R., Molecular dynamics with coupling to an external bath, 1984, J. Chem. Phys., 81, 3684-3690, 10.1063/1.448118

[23] Darden, T., York, D., Pedersen, L., Particle mesh Ewald: An *N*⋅log(*N*) method for Ewald sums in large systems, 1993, J. Chern. Phys., 98, 10089-10092, 10.1063/1.464397

[24] Kühne, T. D., Iannuzzi, M., Del Ben, M., Rybkin, V. V., Seewald, P., Stein, F., Laino, T., Khaliullin, R. Z., Schütt, O., Schiffmann, F., Golze, D., Wilhelm, J., Chulkov, S., Bani-Hashemian, M. H., Weber, V., Borštnik, U., Taillefumier, M., Jakobovits, A. S., Lazzaro, A., Pabst, H., Müller, T., Schade, R., Guidon, M., Andermatt, S., Holmberg, N., Schenter, G. K., Hehn, A., Bussy, A., Belleflamme, F., Tabacchi, G., Glöß, A., Lass, M., Bethune, I., Mundy, C. J., Plessl, C., Watkins, M., VandeVondele, J., Krack, M., Hutter, J., CP2K: An electronic structure and molecular dynamics software package - Quickstep: Efficient and accurate electronic structure calculations, 2020, J. Chem. Phys., 152, 194103, 10.1063/5.0007045

[25] VandeVondele, J., Hutter, J., Gaussian basis sets for accurate calculations on molecular systems in gas and condensed phases, 2007, J. Chem. Phys., 127, 114105, 10.1063/1.2770708

[26] Goedecker, S., Teter, M., Hutter, J., Separable dual-space Gaussian pseudopotentials, 1996, Phys. Rev. B, 54, 1703-1710, 10.1103/PhysRevB.54.1703

[27] Hess, B., Bekker, H., Berendsen, H. J. C., Fraaije, J. G. E. M., LINCS: A linear constraint solver for molecular simulations, 1997, J. Comput. Chem., 18, 1463-1472, 10.1002/(SICI)1096-987X(199709)18:12<1463::AID-JCC4>3.0.CO;2-H

[28] Qin, Y., Han, X., Li, Y., Han, A., Liu, W., Xu, H., Liu, J., Hollow Mesoporous Metal–Organic Frameworks with Enhanced Diffusion for Highly Efficient Catalysis, 2020, ACS Catal., 10, 5973-5978, 10.1021/acscatal.0c01432

[29] He, H.-H., Yuan, J.-P., Cai, P.-Y., Wang, K.-Y., Feng, L., Kirchon, A., Li, J., Zhang, L.-L., Zhou, H.-C., Fang, Y., Yolk–Shell and Hollow Zr/Ce-UiO-66 for Manipulating Selectivity in Tandem Reactions and Photoreactions, 2023, J. Am. Chem. Soc., 145, 17164-17175, 10.1021/jacs.3c03883

[30] Martínez, L., Andrade, R., Birgin, E. G., Martínez, J. M., PACKMOL: A package for building initial configurations for molecular dynamics simulations, 2009, J. Comput. Chem., 30, 2157-2164, 10.1002/jcc.21224

[31] Qin, Y., Han, X., Li, Y., Han, A., Liu, W., Xu, H., Liu, J., Hollow Mesoporous Metal-Organic Frameworks with Enhanced Diffusion for Highly Efficient Catalysis, 2020, ACS Catal., 10, 5973-5978, 10.1021/acscatal.0c01432

[32] Xu, C., He, M., Chen, B., Hu, B., Magnetic S-Functionalized Nanoporous Zr-Metal Organic Frameworks with Defect-Induced Performance in Hg(II) Removal, 2023, ACS Appl. Nano Mater., 6, 17521-17530, 10.1021/acsanm.3c02692

[33] Lu, T., Sobtop, version 1.0 (dev3.2), 2024, http://sobereva.com/soft/Sobtop

[34] Gu, Q., Schiff, E. A., Grebner, S., Wang, F., Schwarz, R., Non-Gaussian Transport Measurements and the Einstein Relation in Amorphous Silicon, 1996, Phys. Rev. Lett., 76, 3196-3199, 10.1103/PhysRevLett.76.3196
